# Association Between Organophosphate Flame Retardant Exposure and Trouble Sleeping: Integrating Epidemiological Evidence with Mechanistic Insights

**DOI:** 10.3390/ijms27041934

**Published:** 2026-02-18

**Authors:** Yifei Guo, Ke Fan, Wenhan Tang, Caoyue Wu, Xin Ni, Tianqi Ling, Linhao Zong, Fei Ma, Miao Guan

**Affiliations:** 1 Jiangsu Key Laboratory for Biodiversity and Biotechnology, College of Life Sciences, Nanjing Normal University, 1 Wenyuan Rd., Nanjing 210023, China; gyf050221@163.com (Y.G.); fk1667298993@163.com (K.F.); 15168706087@163.com (W.T.); 09240314@njnu.edu.cn (C.W.); xin_ni_nnu@163.com (X.N.); 09220235@njnu.edu.cn (T.L.); mafei01@tsinghua.org.cn (F.M.); 2 Ministry of Education Key Laboratory of NSLSCS, Nanjing Normal University, 1 Wenyuan Rd., Nanjing 210023, China

**Keywords:** tributyl phosphate, dibutyl phosphate, trouble sleeping, network toxicology, adverse outcome pathway, *PPARG*, inflammation

## Abstract

Trouble sleeping has become a global public health challenge. However, the relationship between organophosphate flame retardant (OPFR) exposure and trouble sleeping remains unclear. This study integrated epidemiological analysis, network toxicology, molecular docking, molecular dynamics simulations, and adverse outcome pathway (AOP) construction to identify OPFRs linked to trouble sleeping and attempted to elucidate underlying molecular mechanisms. We analyzed cross-sectional data from the U.S. National Health and Nutrition Examination Survey (NHANES 2013–2018) involving 4585 eligible adults. Logistic regression confirmed dibutyl phosphate (DBuP) as significantly correlated with trouble sleeping. Restricted cubic splines (RCSs) revealed a significant non-linear, J-shaped relationship between dibutyl phosphate (DBuP) levels and trouble sleeping. Weighted quantile sum (WQS) analysis determined that DBuP accounted for the majority contribution (58.23%) to the observed effects within exposure mixtures. These findings indicated that DBuP, a metabolite of tributyl phosphate (TnBP), was closely related to trouble sleeping, suggesting that the environmental health risks of TnBP may be jointly contributed to by itself and DBuP. We used network analysis to identify five core target genes (*PPARG*, *MMP9*, *PTGS2*, *APP*, *EGFR*) that interact with DBuP and its parent compound TnBP. Molecular docking predicted binding poses of TnBP and DBuP toward these five core targets; all showed moderate binding affinity (Δ*G* ≤ −5.0 kcal/mol) except MMP9, which exhibited weak binding. Molecular dynamics simulations further supported this putative binding. Enrichment analysis highlighted inflammatory response pathways. Ultimately, we elucidated the process from molecular exposure to trouble sleeping by constructing an AOP framework. In conclusion, we proposed that TnBP and DBuP may contribute to trouble sleeping through multi-target interactions, primarily through PPARG-driven inflammatory dysregulation. These findings suggest a potential link between OPFR exposure and trouble sleeping, providing insights that warrant further mechanistic investigation.

## 1. Introduction

Sleep disorders are a pervasive global health issue. Worldwide, over 16% of the population suffers from insomnia, and nearly 1 billion people are affected by obstructive sleep apnea (OSA) [1,2]. In addition to natural environmental factors such as light and air quality, epidemiological and toxicological evidence suggests a correlation between exposure to environmental pollutants and disrupted sleep patterns [3,4]. Research has linked exposure to metallic elements, including lead, manganese, cadmium, and aluminum, to impaired sleep quality [4]. Furthermore, exposure to synthetic chemicals, such as organophosphate insecticides and bisphenol-A (BPA), has been associated with an increased prevalence of short sleep duration, OSA, and other sleep-related disorders [5,6]. Nevertheless, the variety of environmental pollutants currently known to affect sleep remains relatively limited.

Among numerous pollutants, organophosphate flame retardants (OPFRs) have attracted much attention due to their diverse structures, wide applications, and frequent detection in various environmental media and human samples. Research has shown that the concentration of OPFRs in the atmosphere may have exceeded 800 pg/m^3^, far surpassing many classic persistent organic pollutants (POPs) [7,8]. OPFRs have also been detected globally in aquatic environments, including remote polar regions [8]. These results indicate that OPFRs have spread to global oceans through atmospheric transport and sedimentation processes, becoming emerging pollutants with global distribution characteristics. Due to the wide range of applications of OPFRs, they are pervasively found in various daily items such as building materials, electronic devices, and furniture. In addition to outdoor environmental contamination, modern people usually spend more than 20 h a day in indoor environments [9]. This makes it possible for the public to be exposed to OPFR pollution in their daily lives through various pathways, such as inhaling indoor air, coming into contact with dust containing OPFRs, or ingesting contaminated food and drinking water [10]. The dual exposure indoors and outdoors further increases the risk of OPFR exposure faced by humans. Epidemiological and toxicological studies have demonstrated that OPFRs exhibit various toxic effects in humans, including lipid metabolism disorders, immunotoxicity, impaired reproductive development, and neurotoxicity, with neurotoxicity being the most prominent [11,12,13,14,15,16,17]. For instance, a study in North Carolina found that prenatal exposure to organophosphate esters (OPEs) adversely affected child neurodevelopment [11]. Specifically, higher maternal concentrations of isopropylphenyl phenyl phosphate (ip-PPP) were associated with lower cognitive function in children aged 2–3 years, while exposure to diphenyl phosphate (DPHP) was linked to increased hyperactivity and attention problems in the same age group [11]. Another study, analyzing maternal urine samples collected at 17 weeks of gestation, indicated that prenatal OPFR exposure was associated with an increased risk of attention-deficit/hyperactivity disorder (ADHD) in offspring [13]. Toxicological evidence further supports these findings. Tris(2-butoxyethyl) phosphate (TBEP), a potent neurotoxin, has been shown to alter axonal growth in the secondary motor neurons of zebrafish [16]. Similarly, triphenyl phosphate (TPHP) and tricresyl phosphate (TCP) significantly inhibited acetylcholinesterase activity in zebrafish [15]. In earthworms, exposure to TnBP led to the downregulation of glutaminase expression in the cerebral ganglia, resulting in osmotic imbalance, Ca^2+^ overload, and consequent genetic damage [12]. Additionally, even at relatively low concentrations, tris(2-chloroethyl) phosphate (TCEP) altered the transcription of neurodevelopment-related genes, such as *α1-tubulin*, *gap43*, and *mbp* [14]. The above findings fully demonstrate the neurotoxic effects of OPFRs. Given that OPFRs exhibit neurotoxicity and sleep is under intricate neural regulation, OPFR exposure may plausibly disturb sleep patterns, although the underlying molecular mechanisms remain unclear.

**Figure 1:**
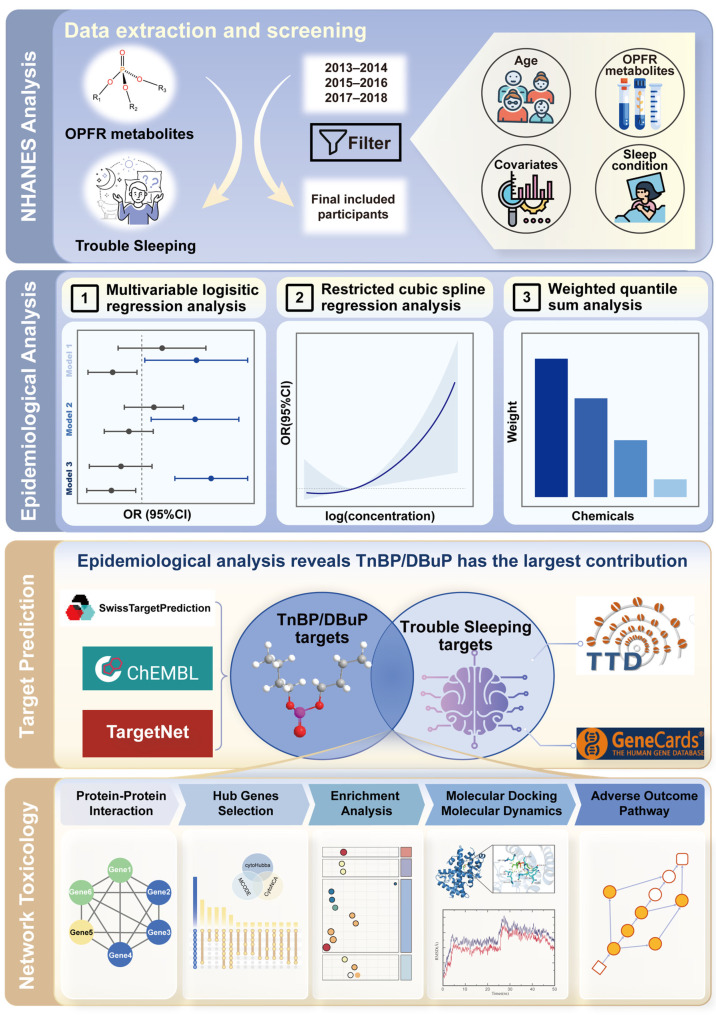
Schematic workflow for deciphering OPFR-linked trouble sleeping: from epidemiological screening to mechanistic exploration, encompassing NHANES analysis, epidemiological analysis, target prediction, and network toxicology.

The National Health and Nutrition Examination Survey (NHANES) employs a stratified, multi-stage probability sampling design to collect health and nutritional information from the U.S. household population, which can be used to study the effects of chemical exposure on health status [18,19]. Its large, representative samples provide high-quality multidimensional data that are pivotal for establishing associations between environmental pollutants and diseases at the population level [20,21]. While sleep disorders encompass a broad spectrum of conditions, this study focuses specifically on trouble sleeping, a self-reported symptom of sleep disorder that is systematically documented in the NHANES database. Sleep disorders are closely associated with the chief complaint of trouble sleeping [22]. The data on the chief complaint of trouble sleeping in NHANES, alongside biomarkers of pollutant exposure in urine and blood, enable the examination of how environmental pollutants may affect sleep health. The adverse outcome pathway (AOP) framework used in this study can construct a coherent system from molecular initiating events to organ-, individual-, and even population-level adverse outcomes [23]. Under this framework, network toxicology offers an interdisciplinary framework. It is widely used in the study of disease mechanisms by integrating bioinformatics, high-throughput data analysis, and genomics to attempt to elucidate molecular mechanisms [24,25,26]. At a more granular level, molecular docking and molecular dynamics simulations predict binding affinity and stability by simulating ligand–protein interactions [27,28]. Thus, integrating network toxicology with molecular docking and molecular dynamics simulations presents an approach for deciphering the toxicological mechanisms of environmental pollutants, effectively connecting epidemiological observations with possible molecular mechanisms (Figure 1).

In this study, we utilized representative cohort data from NHANES and integrated network toxicology with molecular docking and molecular dynamics simulations to investigate the association between OPFRs and trouble sleeping. The objectives of this research were: (1) to identify the OPFR metabolites most closely related to trouble sleeping through epidemiological analysis; (2) to construct a trouble sleeping-related interaction network to identify key targets and pathways; (3) to clarify the complete toxic pathway from early exposure to the final harmful outcome by constructing an AOP. This research will provide novel evidence for the environmental etiology of trouble sleeping and identify potential targets for the early warning and intervention of environmentally related trouble sleeping. It will also offer a scientific basis for environmental health risk assessment and management, thereby contributing to the development of relevant environmental standards and the optimization of public health strategies.

## 2. Results

### 2.1. Characteristics of Participants

Significant differences in various health conditions are observed between individuals with and without trouble sleeping (Table 1). The subgroup proportions of the data release cycle were not statistically significant, whereas the proportions of other subgroups demonstrated significant differences between the trouble sleeping and control groups. Compared with the control group, individuals in the trouble sleeping group had a significantly higher prevalence of asthma, smoking, and hypertension, as well as higher BMI, depression scores, and sedentary time. This indicates that the participants in the trouble sleeping group have a higher comorbidity prevalence and poorer health indicators, reflecting a relatively worse overall health status.

**Figure 2:**
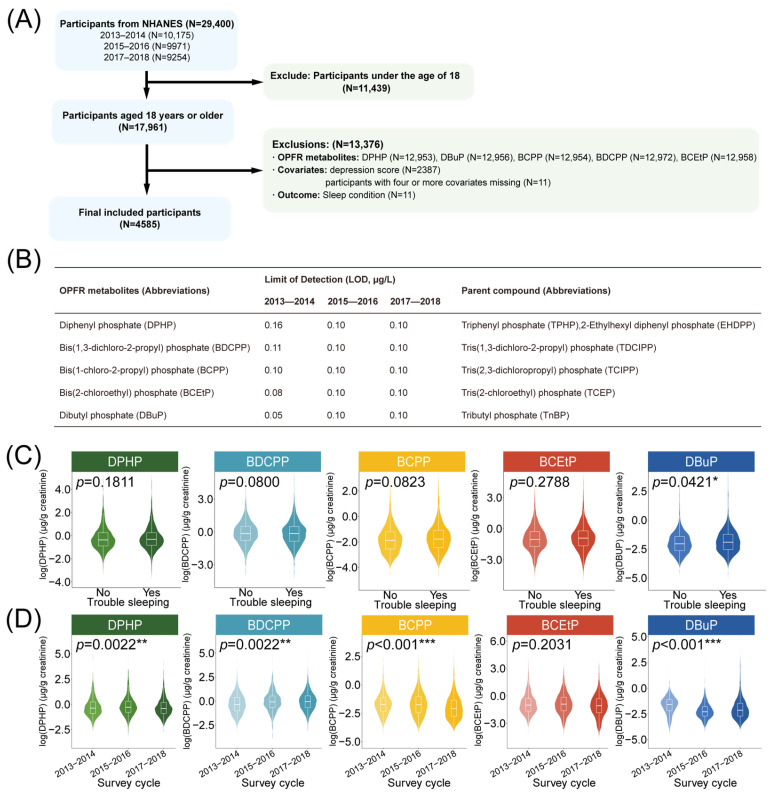
Analytical workflow and distribution of urinary OPFR metabolites in association with sleep status. (**A**) Flowchart for selecting the eligible population in this study. (**B**) Names of urinary OPFR metabolites and parent compounds, limit of detection in different investigation cycles. (**C**,**D**) Violin plots showing the distribution and comparative analysis of urinary metabolite concentrations stratified by sleep status (**C**) and survey cycle (**D**). Asterisks denote statistical significance: * *p* < 0.05, ** *p* < 0.01 and *** *p* < 0.001.

Notably, DBuP was the only metabolite in this study that showed a statistically significant difference in concentration between the trouble sleeping and control groups (Figure 2C). Its geometric mean in the trouble sleeping group was 0.156 ng/(0.01 mg creatinine) and was 0.134 ng/(0.01 mg creatinine) in the control group. Furthermore, with the exception of bis(2-chloroethyl) phosphate (BCEtP), the levels of all other OPFR metabolites varied significantly across survey cycles (Figure 2D). Therefore, we included the survey cycle as a covariate in the model to control for its potential confounding effects.

We conducted Spearman correlation analysis between five OPFR metabolites and trouble sleeping. In addition to bis(1,3-dichloro-2-propyl) phosphate (BDCPP), all other metabolites were positively correlated with trouble sleeping. Notably, DBuP, the metabolite of TnBP, exhibited the strongest positive correlation with trouble sleeping (Figure S1).

### 2.2. Single-Exposure Analysis

**Figure 3:**
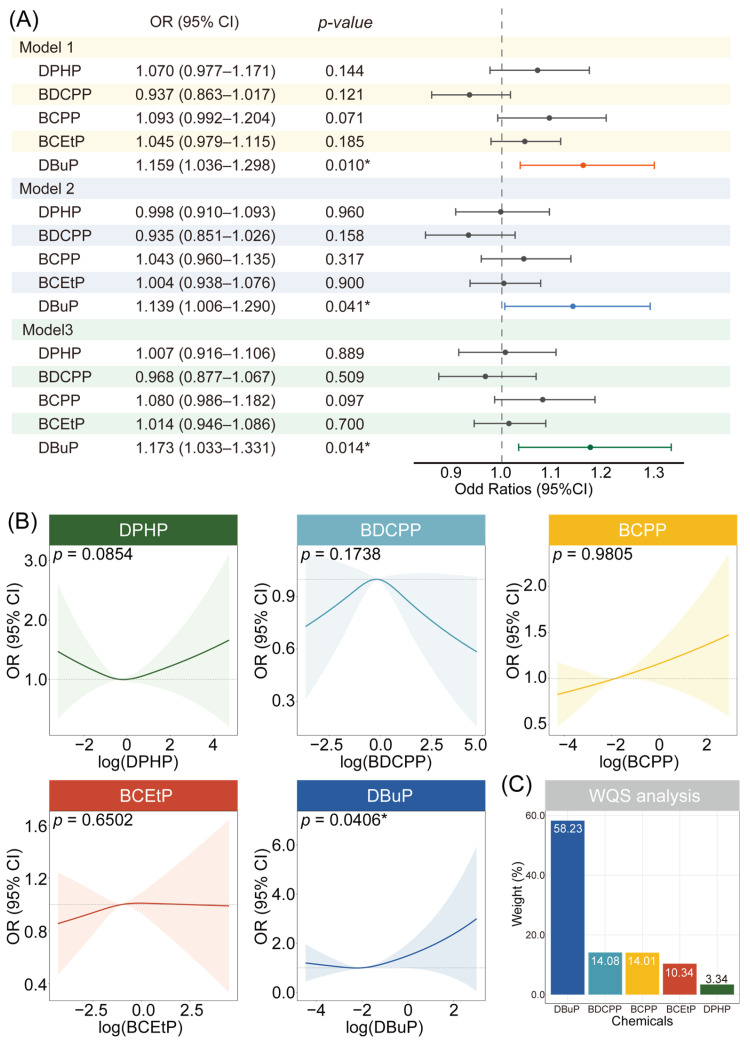
Epidemiological analysis of the relationship between OPFR exposure and trouble sleeping based on multiple methods. (**A**) Association between OPFR exposure and trouble sleeping given by weighted logistic regression. (**B**) Non-linear exposure–response relationships between urinary OPFR metabolite concentrations and trouble sleeping given by RCS regression models. (**C**) The WQS model estimated the contribution proportion of each OPFR metabolite component to trouble sleeping. Asterisks denote statistical significance: * *p* < 0.05.

To evaluate the health risks of exposure to individual OPFR metabolites, binary logistic regression was conducted. Across three progressively adjusted models, a significant positive association was observed between DBuP and trouble sleeping (model 1: OR (95% CI): 1.159 (1.036, 1.298), *p* = 0.010; model 2: 1.139 (1.006, 1.290), *p* = 0.041; model 3: 1.173 (1.033, 1.331), *p* = 0.014) (Figure 3A). The other four OPFR metabolites, DPHP, BDCPP, bis(1-chloro-2-propyl) phosphate (BCPP), and BCEtP, did not demonstrate any significant effects. We further employed the RCS regression model to estimate the potential non-linear exposure–response relationship between OPFR metabolites and trouble sleeping, with adjustment for all 11 covariates. A significant non-linear “J-shaped” association was identified (*p* = 0.0406) for DBuP. DPHP, BDCPP, BCPP, and BCEtP did not demonstrate any significant associations with trouble sleeping. The risk of trouble sleeping initially decreased slightly with increasing DBuP concentrations, reaching a minimum OR of 0.994 at a DBuP level of 0.107 μg/g creatinine (log-transformed value: −2.235). Beyond this inflection point, the risk increased substantially with rising DBuP concentrations (Figure 3B). Sensitivity analysis using urinary creatinine as an additional covariate yielded similar results (Figure S2).

### 2.3. Mixture Co-Exposure Analysis

To evaluate the joint effect of OPFRs co-exposure on trouble sleeping, we applied WQS analysis. The model revealed a statistically significant positive association between the OPFR mixture and trouble sleeping, with an OR of 1.066 (95% CI: 1.016, 1.119; *p* = 0.010) per increment in the mixture index. Decomposition of the mixture index identified DBuP as the most influential compound, accounting for 58.23% of the total weight. The weights of BDCPP, BCPP, BCEtP, and DPHP were 14.08%, 14.01%, 10.34%, and 3.34%, respectively (Figure 3C). This indicates that DBuP plays a dominant role in the occurrence of trouble sleeping under real-world mixed OPFR exposure scenarios.

### 2.4. Identification of Cross-Target and Protein–Protein Interaction (PPI) Network Construction for TnBP/DBuP-Induced Trouble Sleeping

As both single-exposure analysis and mixture co-exposure analysis revealed that DBuP (metabolite of TnBP) was the most important component associated with trouble sleeping, we further conducted a network toxicology study on TnBP and DBuP.

**Figure 4:**
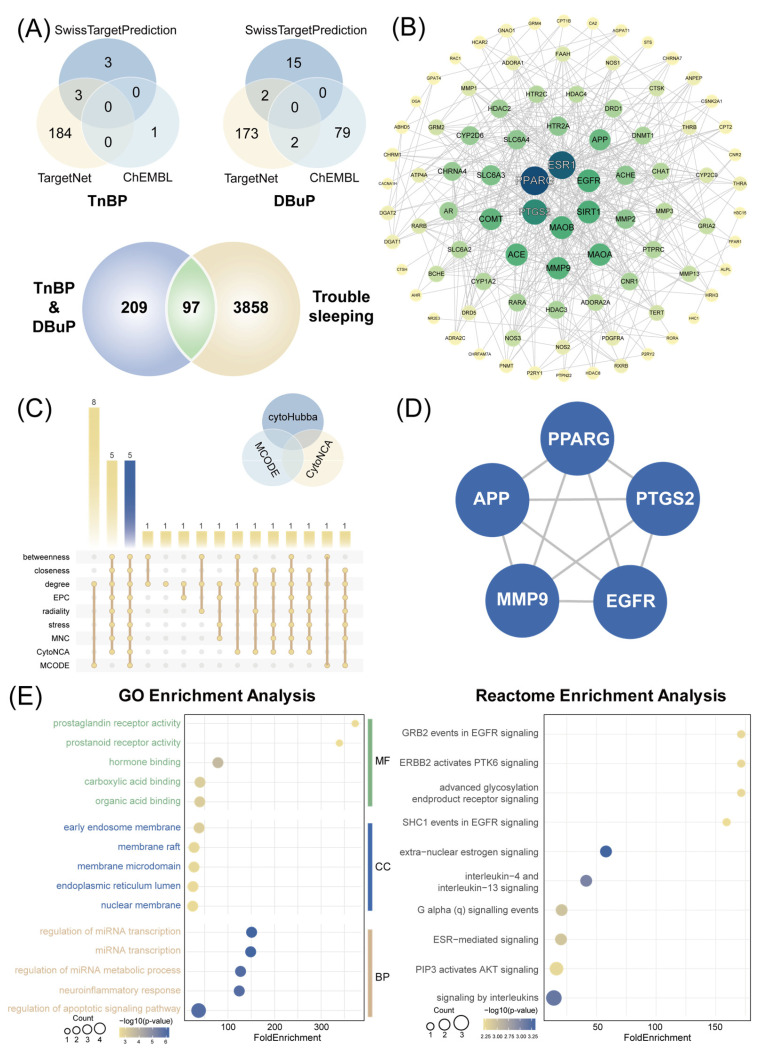
Results of network toxicology analysis. (**A**) Venn diagram showing the overlap among 191 TnBP targets, 271 DBuP targets, and trouble sleeping-related genes (97 shared targets). (**B**) PPI network of overlapping genes with node attributes reflecting topological importance. (**C**) Overlapping genes identified via seven computational algorithms. (**D**) Integrated PPI network of hub genes derived from multi-algorithm consensus. (**E**) GO and Reactome pathway enrichment analyses.

We employed a multi-database integration strategy for target identification. A total of 3955 candidate genes associated with trouble sleeping were retrieved from GeneCards and Therapeutic Target Database. We identified 191 potential targets of TnBP and 271 potential targets of DBuP from the ChEMBL, SwissTargetPrediction, and TargetNet databases. Given that DBuP is a metabolic derivative of TnBP, we consolidated the target profiles of both compounds, resulting in a combined set of 306 unique targets for subsequent analyses. A total of 97 overlapping targets were identified at the intersection, representing 31.70% of the 306 potential targets, which may be implicated in TnBP/DBuP-induced trouble sleeping (Figure 4A).

The PPI network was constructed with STRING, and a layered network was visualized in Cytoscape (Figure 4B). Hub genes were localized at the center, rendered in blue, while intermediate nodes in the adjacent layer were assigned green, and peripheral nodes at the outermost region were depicted in yellow. *PPARG*, *PTGS2*, *EGFR*, and other genes may act as core regulators in TnBP/DBuP-induced trouble sleeping.

### 2.5. Hub Gene Screening and Functional Enrichment Analysis

The top 15 candidate genes from each of the seven cytoHubba algorithms, CytoNCA and MCODE were extracted. Intersection analysis identified five high-confidence overlapping targets, visualized via an UpSet plot (Figure 4C, Table S2). A systematic integration of the top-ranked genes from three distinct network plugins (cytoHubba, MCODE, and CytoNCA) was performed to refine the hub gene set. This process yielded a final set of five hub genes (*PPARG*, *MMP9*, *PTGS2*, *APP*, *EGFR*) central to the trouble sleeping-associated network. The high-confidence PPI network exhibited a dense interconnectivity among the hub genes, forming critical interaction modules consistent with their coordinated role in driving the trouble sleeping response (Figure 4D). After calculation, the degree values of the five hub genes were identical, resulting in uniform node coloring in the network visualization.

To systematically characterize the functional profile of the target gene set, we performed Gene Ontology (GO) and Reactome pathway enrichment analysis. The bubble plot quantified enrichment strength through color gradients (−log_10_*p*-values) and bubble areas (gene counts). Fold enrichment serves as a metric to assess the degree of target gene overrepresentation in a given pathway. The fold enrichment suggests that TnBP/DBuP significantly perturbs this pathway, implying a potential role for them in the mechanism underlying trouble sleeping. Pathways including nuclear membrane, regulation of apoptotic signaling pathway, prostanoid receptor activity, and neuroinflammatory response were significantly enriched. Furthermore, EGFR-associated signaling pathways were recurrently observed in the Reactome enrichment analysis results, including SHC1 events in EGFR signaling and GRB2 events in EGFR signaling. Additionally, pathways like signaling by interleukins, interleukin-4, and interleukin-13 signaling, which were closely related to inflammatory response, were also significantly enriched (Figure 4E, Tables S3–S6).

### 2.6. Molecular Docking and Molecular Dynamics Simulation of TnBP and DBuP to the Hub Genes

**Figure 5:**
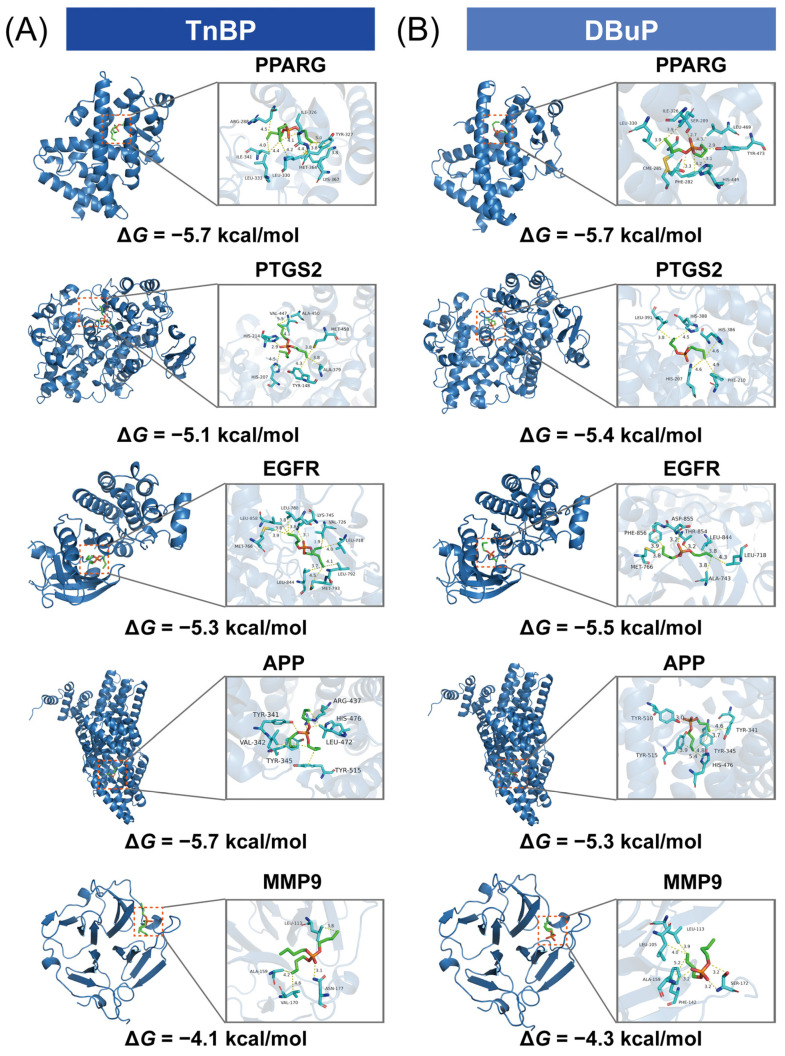
Molecular docking analysis of TnBP and DBuP with key target proteins. (**A**) The three-dimensional conformational diagram of the molecular docking results of TnBP with five core target proteins (PTGS2, PPARG, APP, EGFR, MMP9) and their calculated binding free energies (kcal/mol). (**B**) The three-dimensional conformational diagram of DBuP docking with the same five target proteins and its corresponding calculated binding free energy. Color scheme: Orange for phosphorus; red for oxygen; green for carbon chains; blue for protein or amino acid.

To systematically evaluate the interaction strength with potential toxic targets, we simulated the binding conformations and determined the affinities of TnBP and DBuP with five core targets (PTGS2, PPARG, APP, EGFR, and MMP9) (Figure 5, Table S7). A binding free energy (Δ*G*) value below −5.0 kcal/mol indicates a moderate ligand–protein binding affinity [29]. The results revealed that the binding free energy of TnBP with targets ranged from −4.1 to −5.7 kcal/mol, while that of DBuP with the targets ranged from −4.3 to −5.7 kcal/mol. Among the five identified core targets, PTGS2, PPARG, APP, and EGFR exhibited Δ*G* values below −5.0 kcal/mol upon interaction with TnBP/DBuP, indicative of moderate ligand–protein binding. The binding ability of MMP9 to TnBP and DBuP was weak. These results suggest that PTGS2, PPARG, APP, and EGFR, with their moderate binding affinities, are the most likely direct mediators of TnBP/DBuP-induced trouble sleeping. In contrast, the weak binding observed for MMP9 implies its potential role may be indirect or auxiliary in the pathological process.

**Figure 6:**
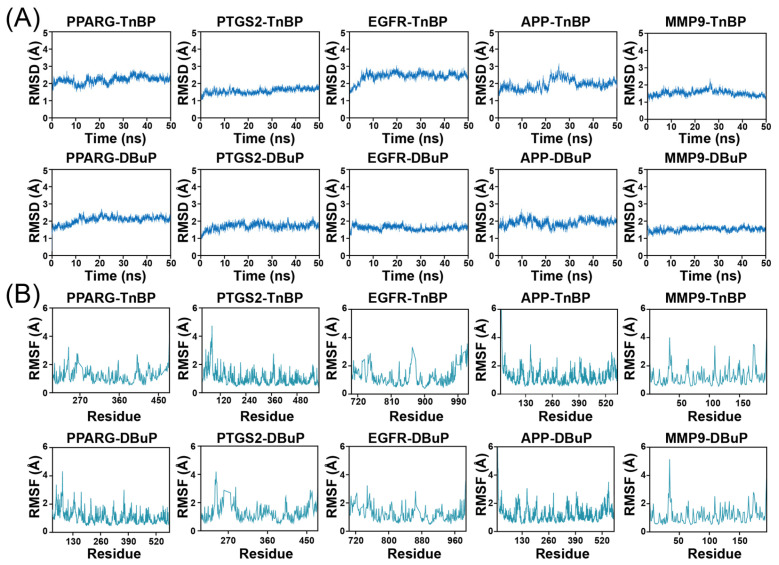
Molecular dynamics simulation of TnBP and DBuP with key target proteins. (**A**) Root mean square deviation (RMSD) and (**B**) root mean square fluctuation (RMSF) profiles derived from molecular dynamics simulation analysis.

To further validate the binding of the five key proteins with TnBP and DBuP, molecular dynamics simulations were performed. Root mean square deviation (RMSD) was employed to assess the conformational stability of the protein–ligand complexes and the deviation of atomic positions from their initial coordinates. Most receptor–ligand complexes reached equilibrium after approximately 5 ns, with RMSD values exhibiting small fluctuations within around 1 Å (Figure 6A). Root mean square fluctuation (RMSF) was used to evaluate the flexibility of amino acid residues. The RMSF values of each complex remained below 4 Å in most cases, indicating low overall residue flexibility and high conformational stability (Figure 6B). Hydrogen bonding interactions played a critical role in binding. Persistent and dynamically exchanging hydrogen bonds were observed in all complexes, contributing to stable ligand association (Figure S3A). The dynamic nature of these interactions suggests intrinsic flexibility within the binding sites. Radius of gyration (Rg) and solvent-accessible surface area (SASA) exhibited only minor fluctuations across all complexes, reflecting stable and compact protein–ligand binding (Figure S3B,C). The x-axis of the free energy spectrum (FEL) of the complex is RMSD (nm), and the y-axis is Rg (nm). Red regions indicate high free energy, while blue denotes low free energy. Each complex exhibited at least one energy minimum within the predefined free energy range, confirming the attainment of thermodynamically stable conformational states (Figure S3D).

### 2.7. Establishment of the TnBP/DBuP-Induced Trouble Sleeping AOP Framework

**Figure 7:**
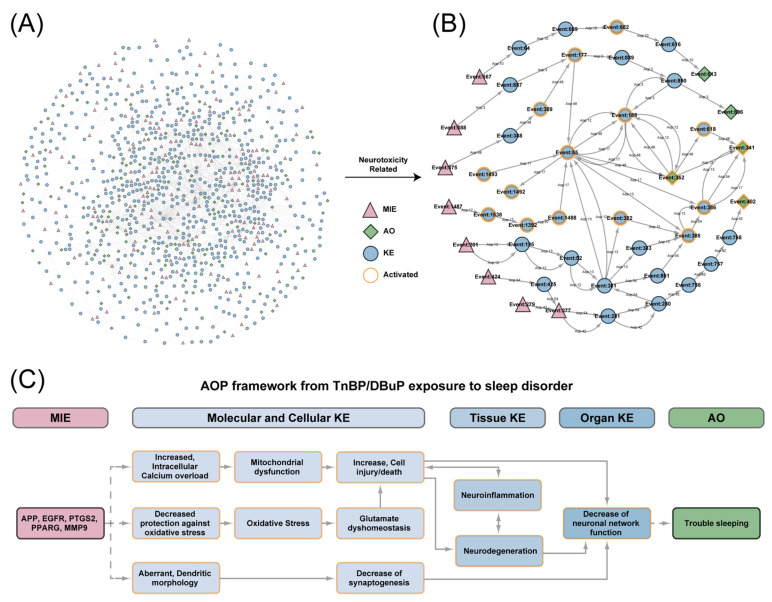
Construction of the AOP framework for TnBP/DBuP-induced trouble sleeping. (**A**) Global AOP network encompassing all human-applicable AOPs. (**B**) Neurotoxicity-specific AOP network. (**C**) Final constructed AOP framework. Molecular initiating events (MIEs) are marked in red, key events (KEs) in blue, and adverse outcomes (AOs) in green. Activated events are highlighted with orange outlines. Solid lines represent key event relationships (KERs) with evidence and adjacency as documented in AOP-Wiki.

The global AOP network, encompassing all human-applicable AOPs, is illustrated in Figure 7A. From this network, we extracted a neurotoxicity-specific AOP network based on its status and neurotoxicity relevance (Figure 7B). Detailed information regarding the neurotoxicity AOP network is provided in Table S8. By analyzing the consecutive activated events within the neurotoxicity AOP network, we identified KEs from molecular to organ level as the core components of the final AOP framework. As depicted in Figure 7C, TnBP/DBuP may activate molecular initiating events (MIEs) involving five hub genes, leading to increased cell injury/death and decreased synaptogenesis at the cellular level. This, in turn, triggers neuroinflammation and neurodegeneration at the tissue level, resulting in impaired neuronal network function and ultimately trouble sleeping.

## 3. Discussion

By analyzing NHANES data from 2013 to 2018, this study identified DBuP, the metabolite of TnBP, as the OPFR metabolite most strongly associated with trouble sleeping. Additionally, network toxicology analyses identified five hub genes (*PTGS2*, *PPARG*, *APP*, *EGFR*, and *MMP9*) in TnBP/DBuP-induced trouble sleeping. Furthermore, molecular docking and molecular dynamics simulations revealed that PTGS2, PPARG, APP, and EGFR are the moderate-affinity binding targets for TnBP and DBuP, providing structural biological evidence for the molecular mechanism of TnBP/DBuP-induced trouble sleeping.

*PPARG* is a nuclear receptor protein that plays a key role in gene expression, metabolic regulation, and inflammatory response [30]. Previous studies have shown that *PTGS2* is one of the target genes of *PPARG*. *PPARG* activation can inhibit *PTGS2* expression, thereby reducing prostaglandin E2 (PGE2) synthesis and inhibiting inflammation and tumor growth [31]. The tight binding between TnBP/DBuP and PPARG may interfere with this process and promote the occurrence of inflammatory reactions.

*PTGS2* is a critical mediator of inflammatory signaling. Functioning as the rate-limiting enzyme in prostaglandin biosynthesis, PTGS2 catalyzes the conversion of arachidonic acid to PGE2 and other eicosanoids. PGE2 is a key lipid mediator in inflammation [32]. Beyond its canonical role in eicosanoid production, *PTGS2*-derived PGE2 modulates inflammatory responses through the nuclear factor-kappa B (NF-κB) signaling cascade and amplifies the inflammatory cascade by potentiating the expression of proinflammatory cytokines, including interleukin-6 (IL-6) and tumor necrosis factor-alpha (TNF-α) [33,34]. Research has shown that the release of inflammatory factors can activate the epidermal growth factor receptor (EGFR) [35,36]. Meanwhile, phosphorylation and activation of EGFR can be induced by other epidermal growth factors [37]. Src Homology and Collagen1 (SHC1) binds to Tyr-1086 and Tyr-1114 on EGFR through its C-terminal PTB domain [38]. After binding to activated EGFR through the PTB domain, SHC mainly interacts with GRB2-SOS complexes and plays an important role in the Ras signaling pathway [39]. Son of sevenless (SOS) can catalyze the conversion of the membrane-anchored protein RAS from an inactive GDP-bound state to an active GTP-bound state. Activated RAS triggers the mitogen-activated protein kinase (MAPK) cascade reaction [40]. MAPK further amplifies the TNF-α signal through positive feedback, thereby enhancing the activation of NF-κB [41,42]. The p65 subunit of NF-κB can inhibit nuclear factor erythroid 2-related factor 2 (NRF2) expression through competitive coactivation [43]. As a key transcription factor in antioxidant response, the downregulation of NRF2 expression directly leads to a decrease in cellular antioxidant capacity, which in turn leads to oxidative stress [44]. This forms a molecular bridge from inflammation to oxidative stress. The downregulation of NRF2 expression directly weakens the antioxidant defense ability of cells, which corresponds to the first KE of decreased antioxidant capacity in the AOP framework constructed in this study. Subsequently, the decrease in antioxidant capacity led to a significant increase in intracellular oxidative stress levels (the second KE) (Figure 7C). The AOP framework suggests that these key events can lead to cell damage or death, causing neuroinflammation, resulting in neural network dysfunction, and ultimately leading to trouble sleeping.

Enrichment analysis in this study provides preliminary direct support for the proposed molecular cascade (Figure 4E). The enrichment of pathways such as neuroinflammatory response and interleukin-4 and interleukin-13 signaling enhances the core role of extensive neuroinflammatory response. The significant enrichment of GRB2 events in EGFR signaling and SHC1 events in EGFR signaling can support the hypothesis that EGFR is activated by the inflammatory environment after TnBP exposure, and binds with SHC1, GRB2, and SOS to activate downstream RAS/MAPK pathways. In summary, these findings confirm that inflammatory signals flow through the SHC1/GRB2/SOS/RAS/MAPK cascade and continuously amplify in a coherent signal network, leading to neural network damage and endangering sleep health.

*APP* is critically implicated in the pathogenesis of neurodegenerative diseases, most notably Alzheimer’s disease [45]. As a primary driver of amyloid-β (Aβ) genesis, elevated *APP* expression promotes Aβ overproduction. The subsequent accumulation of Aβ peptides can induce hyperexcitability in local interneurons [46], ultimately culminating in neurotoxicity. In GO enrichment analysis, pathways like the regulation of presynapse assembly, neurotransmitter uptake, and neurotransmitter transport are significantly enriched (Figure 4E). This result suggests that APP has an impact on these pathways, interfering with the normal function of synapses. Additionally, the AOP framework exhibits key events such as abnormal dendritic morphology and reduced synaptic formation, which directly lead to neural network dysfunction and interfere with sleep health (Figure 7C). Pathways related to *MMP9*, like the negative regulation of cation transmembrane transport and negative regulation of monoatomic ion transport, are significantly enriched in the GO enrichment analysis. This result is closely related to the key event of increased intracellular calcium overload in the AOP framework. TnBP and DBuP may interfere with the transmembrane transport of calcium ions by affecting ion channels on the cell membrane, leading to mitochondrial dysfunction, cell damage or death, and ultimately potentially pointing to neural network damage and trouble sleeping.

Collectively, all of the above results lead us to propose the following hypothesis. TnBP and DBuP first tightly bind and interfere with the normal function of nuclear receptor *PPARG*, relieving its transcriptional inhibition of downstream target gene *PTGS2*, leading to an increased expression of *PTGS2* and synthesis of PGE2. PGE2, as a key inflammatory mediator, activates NF-κB signaling and promotes the release of pro-inflammatory factors such as TNF-α and IL-6. It then activates the epidermal growth factor receptor (EGFR) and its downstream SHC1/GRB2/SOS/RAS/MAPK cascade, forming an inflammatory positive feedback amplification loop and continuously enhancing NF-κB activity. Activated NF-κB further reduces the expression of antioxidant transcription factor NRF2 through competitive inhibition, leading to a decrease in cellular antioxidant capacity and an increase in oxidative stress levels. Ultimately, under the combined drive of inflammation and oxidative stress, the disruption of the neural microenvironment triggers neuroinflammation, neural network damage, and functional abnormalities, leading to trouble sleeping. TnBP and DBuP may also disrupt synaptic function and neural health by promoting *APP* expression and Aβ accumulation, while simultaneously interfering with calcium ion transport through pathways associated with *MMP9*, leading to intracellular calcium overload and cellular damage. In conclusion, neuroinflammation, oxidative stress, Aβ-related synaptic toxicity, and calcium imbalance collectively lead to neural network dysfunction, resulting in trouble sleeping.

This study begins with epidemiological observations and employs network toxicology for broad-spectrum target screening and mechanistic hypothesis building. Molecular docking is subsequently utilized to provide preliminary structural-level interaction evidence. These multi-layered insights are ultimately consolidated within a cohesive AOP framework, which systematically links molecular initiating events to the adverse health outcome. This integrated methodology advances the mechanistic understanding of TnBP’s potential toxicity while simultaneously providing a transferable template for investigating associations between environmental contaminants and complex diseases. It is critical to acknowledge, however, that the present findings remain largely derived from computational predictions and epidemiological correlations. Consequently, definitive causal relationships and the precise nature of the molecular interactions require further experimental validation. Future studies should prioritize in vitro binding assays and longitudinal in vivo investigations to verify the proposed signaling cascade and to establish a causative link between TnBP exposure and trouble sleeping.

## 4. Materials and Methods

### 4.1. Study Population

The survey data from NHANES were analyzed to investigate the exposure–response relationship between urinary OPFR metabolite concentrations and sleep status. All datasets utilized in this study are publicly accessible through the NHANES website (http://www.cdc.gov/nchs/nhanes.htm, accessed on 28 August 2025). Specifically, three cross-sectional survey waves (2013–2014, 2015–2016, and 2017–2018) were merged for analysis. Participants were included following the criteria outlined in Figure 2A, resulting in a total of 4585 adults aged 18 years and older.

### 4.2. Exposure Variables

Existing studies have demonstrated that urinary levels of these metabolites are valid biomarkers for assessing human exposure to their parent OPFRs (Figure 2B) [47]. Therefore, urinary OPFR metabolite concentrations were selected as exposure variables, with a total of five metabolites evaluated: DPHP, BDCPP, BCPP, BCEtP, and DBuP. Quantification of these urinary metabolites was performed using ultra-performance liquid chromatography coupled with electrospray tandem mass spectrometry (UPLC-MS/MS). Detailed analytical protocols, including quality-control (QC) materials, limit of detection (LOD), and inter-batch coefficients of variation, are documented in the NHANES laboratory procedures (Figure 2B). Concentrations below the LOD were replaced with LOD/$\sqrt{2}$, consistent with standard NHANES practice. Only participants with detectable values or valid LOD substitutions for all five metabolites were included in the analysis.

To adjust for physiological variations in urine dilution, renal function, and metabolism, OPFR metabolite concentrations were normalized to urinary creatinine levels [47]. The normalized values are expressed as ng/(0.01 mg creatinine) to facilitate inter-individual comparisons. Additionally, these creatinine-adjusted concentrations were natural log-transformed before statistical modeling to approximate normality and reduce skewness.

### 4.3. Outcome Variables

Sleep status was assessed using the health questionnaire included in NHANES. Trouble sleeping was defined based on participants’ responses to the question “Ever told doctor had trouble sleeping”. Affirmative answers (“Yes”, coded as 1) indicated the presence of trouble sleeping, while negative responses (“No”, coded as 0) indicated its absence. Participants who answered “Don’t know” (coded as 7) or “Refused” (coded as 9) were excluded from the analysis as missing values.

### 4.4. Covariates

To estimate the association between OPFR exposure and trouble sleeping while accounting for potential confounders, our models included 11 covariates. The covariates included age, gender, race, smoking status, vigorous recreational activity, hypertension, asthma, depression, cycle, body mass index (BMI), and sedentary time. Age was stratified into three categories: 18–49, 50–79, and ≥80 years. Participants were classified as female or male. Race was grouped into six categories: Mexican American, Other Hispanic, Non-Hispanic White, Non-Hispanic Black, Non-Hispanic Asian, and Other Race. Smoking status was determined as “yes” for “Smoked at least 100 cigarettes in life” or “no”. Vigorous recreational activities, hypertension, and asthma were determined as “yes” or “no”. Survey cycle included 2013–2014, 2015–2016, and 2017–2018. Nine-item Patient Health Questionnaire (PHQ-9) scores were selected as the measurement of depressive symptoms. BMI and sedentary time were also continuous variables. After testing, the distribution of the four continuous variables (depression score, BMI, and sedentary time) in both the trouble sleeping group and the control group did not follow a normal distribution. As a result, differences in covariates by sleep status were assessed using the Wilcoxon rank-sum test for continuous variables and chi-square tests for categorical variables.

### 4.5. Statistical Analyses

This study utilized data from three cycles of NHANES. To account for its complex survey design, including oversampling, non-response, and population stratification, and to generate estimates representative of the non-institutionalized U.S. civilian population, all analyses incorporated the recommended examination sample weights (Table S1). Specifically, following the NHANES analytical guidelines, we constructed a combined weight across the three survey cycles (2013–2014, 2015–2016, and 2017–2018) by integrating their respective cycle-specific subsample weights: WTSSCH2Y (Surplus specimen C 13–14 2 year weights), WTSSBI2Y (Surplus specimen B 15–16 2 year weights), and WTSB2YR (Standardized Biomarkers Subsample Weight for 2-Year Cycle). The detailed operation was to adjust the weight by dividing it by the number of cycles included (3 cycles in this study), based on the official recommendations of NHANES, so that it can represent the overall situation of the target population in the multi-year merged sample. This processing step is crucial for generating unbiased and nationally representative statistical data. Meanwhile, we used the “SDMVPSU (Masked variance pseudo-PSU)” and “SDMVSTRA (Masked variance pseudo-stratum)” provided in the “Demographic Variables and Sample Weights” file as the Primary Sampling Unit (PSU) and stratum, respectively. Before formal analysis, we cleaned up and prepared the data strictly. In order to ensure the effectiveness of the multiple imputation method and avoid the instability of the imputation model caused by too many missing covariate values, we excluded samples with four or more missing covariate values. At the same time, the depression score, as a potentially important confounding variable that this study focuses on, was missing too much. Direct imputation may affect the accuracy of the results. We therefore removed samples with missing depression scores. Subsequently, we used multiple imputation to fill in missing covariate values in the remaining data. We set the number of iterations to 20 and generated 5 datasets to perform multiple imputation on missing values of covariates [48]. Finally, we removed samples with missing outcome variables (trouble sleeping) or urinary concentrations of OPFR metabolites. All subsequent statistical analyses were based on the imputed complete dataset. The R (version 4.5.0) “mice” and “mitools” packages were employed for multiple imputation.

In order to control confounding factors and improve model accuracy, we established three gradually adjusted models. Weighted logistic regression was applied to examine the association between exposure to each chemical and the risk of trouble sleeping. Model 1 was unadjusted. Model 2 was adjusted for age, gender, race, BMI, hypertension, and data release cycle to correct for the impact on population characteristics and basic health status. Model 3 was further adjusted for sedentary time, vigorous recreational activities, depression, asthma, and smoking status to further correct the impact of lifestyle habits and diseases on the analysis. The R (version 4.5.0) “survey” package was employed to weight the data and adjust for stratification. We used the Wald test to assess the association between individual chemical exposure and trouble sleeping. Restricted cubic splines (RCSs) were employed to characterize the dose–response relationship between individual urinary OPFR metabolite concentrations and the risk of trouble sleeping. The “rms” package was used to fit the logistic regression model and construct the restricted cubic spline.

For the effects of mixture exposure, weighted quantile sum (WQS) regression (bootstrap = 1000) was employed to explore the overall impact of OPFR metabolite mixtures on trouble sleeping. We used the R package “gWQS” to calculate weights for individual chemicals and the corresponding odds ratios (ORs). The model was adjusted for 11 covariates described above (Table 1).

### 4.6. Collection of Targets of Two Compounds (DBuP and TnBP) and Trouble Sleeping

Based on the epidemiological findings from NHANES, the metabolite DBuP, which is a primary metabolite of TnBP, exhibited the strongest association with trouble sleeping in both single-exposure and mixture co-exposure models. Therefore, we selected DBuP and its parent compound TnBP for subsequent network toxicology analysis to explore their potential mechanisms of inducing trouble sleeping.

The molecular structures of DBuP and TnBP were retrieved in Structure-Data File (SDF) format from the PubChem database (https://pubchem.ncbi.nlm.nih.gov, accessed on 8 November 2025). To predict potential protein targets of the two target analytes, a multi-database screening strategy was employed, integrating data from ChEMBL (https://www.ebi.ac.uk/chembl/, accessed on 8 November 2025), PharmMapper (https://www.lilab-ecust.cn/pharmmapper/index.html, accessed on 8 November 2025), SwissTargetPrediction (https://swisstargetprediction.ch/, accessed on 8 November 2025), and TargetNet (http://targetnet.scbdd.com/, accessed on 8 November 2025) [49,50,51]. All database queries were conducted using version-controlled data as of 11 October 2025, with biological filters restricted to *Homo sapiens* applied during the predictive modeling process. Subsequently, the target protein results for the two analytes from the four databases were merged to form a union set, and duplicate entries were eliminated to serve as the potential targets for the two substances. For a comprehensive analysis of trouble sleeping-related associations, seven terms, including “trouble sleeping”, “sleep disorder”, “sleep disturbance”, “sleep dysfunction”, “insomnia”, “parasomnia”, and “dyssomnia”, were queried in the GeneCards database (https://www.genecards.org/) and Therapeutic Target Database (TTD) (https://ttd.idrblab.cn/) on 5 February 2026 [52]. In the GeneCards database, targets with a *Relevance score* less than 0.4 were excluded.

### 4.7. Construction of Protein–Protein Interaction (PPI) Networks and Identification of Hub Genes

After integrating the aforementioned target genes and removing redundancies, the combined set of trouble sleeping-associated targets specific to DBuP and TnBP was identified as the candidate gene set underlying the two-target compound-induced trouble sleeping. Using these genes, a protein–protein interaction (PPI) network was constructed via the STRING database (https://cn.string-db.org/, accessed on 5 February 2026) with parameters set to “*Homo sapiens*” as the organism and a medium confidence interaction score threshold of 0.4. Subsequently, network attributes, including edges, nodes, and degree values, were extracted for subsequent analysis.

To evaluate the topological properties of the identified targets, the cytoHubba plugin within the Cytoscape software (version 3.10.0) was employed. Seven distinct algorithms were utilized, including betweenness centrality, closeness centrality, degree centrality, Edge Percolation Component (EPC), Maximum Neighborhood Component (MNC), radiality, and stress. These algorithms were selected to provide a comprehensive analysis of network characteristics, focusing on node roles as bridges, their proximity to other nodes, connectivity count, the importance of edges in network connectivity, local clustering, centrality, and network flow stress, respectively [53,54]. The top 15 genes ranked by each algorithm were intersected to identify overlapping candidates. Additionally, the CytoNCA and MCODE plugins were applied to further prioritize genes based on their scores. The steps of using the CytoNCA plugin for screening are calculating the median of each parameter and selecting candidate genes with scores above the median for each parameter. The MCODE plugin generates critical subnetworks during runtime, from which genes can be screened. Finally, the intersection of key genes identified by these three plugins (cytoHubba, CytoNCA, and MCODE) was determined, and the shared genes were designated as “hub genes”, representing the most critical targets involved in trouble sleeping elicited by DBuP and TnBP.

### 4.8. Functional Enrichment Analysis for Overlapping Genes and Hub Genes

Functional enrichment analysis of overlapping genes and hub genes was performed using the “clusterProfiler” and “ReactomePA” packages in R, which utilize Gene Ontology (GO) terms and Reactome pathway databases. For GO enrichment analysis, we utilized the “org.Hs.eg.db” database with the significance thresholds set at *p* < 0.05 and *q* < 0.05 to control for multiple testing. For Reactome pathway enrichment analysis, the organism was specified as “hsa” (*Homo sapiens*), with a significance cutoff of *p* < 0.05. These parameters ensured robust and biologically relevant enrichment analysis outcomes. This systematic approach provides critical insights into the biological processes and molecular mechanisms associated with the identified targets.

### 4.9. Molecular Docking and Molecular Dynamics Simulation of DBuP and TnBP with Hub Genes

To investigate the molecular interactions between the test compounds and targets related to trouble sleeping, a detailed molecular docking study was conducted. Crystal structures of the key target proteins were obtained from the RCSB Protein Data Bank (PDB). Meanwhile, the 3D structural data of DBuP and TnBP were acquired from the PubChem database and converted to PDBQT format to enable flexible ligand docking. The grid box was defined using AutoDockTools (version 1.5.7). Molecular docking was performed using AutoDock Vina, with 9 predicted docking poses generated. The conformation with the lowest binding free energy was selected as the optimal result for further analysis. Receptor–ligand interactions (including hydrogen bonding, hydrophobic interactions, π-π stacking, etc.) were analyzed using Discovery Studio 2021 Client. Finally, visualization was performed using PyMOL (version 3.10) [55,56].

In this study, a molecular dynamics simulation was performed using GROMACS 2022. The force field parameters were generated by the pdb2gmx tool of GROMACS [57]. Sobtop 1.0 (dev3.1) software was used to generate the topology file of gaff2 force field according to the ligand structure. The ligand was assigned charge by resp method to ensure that the charge distribution conforms to the physicochemical characteristics [58]. The system was solvated in a cubic box and maintained at 310 K and 1.0 bar during 50 ns simulations [28]. Root mean square deviation (RMSD), root mean square fluctuation (RMSF), hydrogen bonds (hbonds), radius of gyration (Rg), solvent accessible surface area (SASA), and free energy landscape were analyzed using GROMACS tools to evaluate the stability, structural changes, and solvent effects of the system [59].

### 4.10. AOP Framework Construction

We developed an Adverse Outcome Pathway (AOP) framework to elucidate the toxic mechanisms underlying TnBP/DBuP-induced trouble sleeping. An AOP formally describes a sequence of events starting from molecular initiating events (MIEs), proceeding through key events (KEs) at molecular, cellular, and tissue levels, and culminating in adverse outcomes (AOs) at organ, organism, or population levels, with key event relationships (KERs) linking these events. Briefly, we downloaded all available AOP information from the AOP-Wiki and filtered for human-relevant AOPs based on species applicability. Subsequently, we constructed a global AOP network using Cytoscape. From this network, we identified high-quality human neurotoxicity AOPs that were both related to neurotoxicity and endorsed in status [60], extracting these to form a neurotoxicity AOP network while retaining only adjacent KERs within it. Next, based on the GO enrichment analysis results of the screened hub genes, we annotated the events in the neurotoxicity AOP network, with significantly enriched events considered activated [61]. Finally, we extracted consecutive activated events from this sub-network as key components to construct the final AOP framework.

## 5. Conclusions

This study revealed that DBuP, a metabolite of TnBP, represented the most critical OPFR metabolite linked to trouble sleeping in this study, displaying a non-linear exposure–response relationship. Utilizing a multi-layered computational approach, we systematically identified five hub genes. Molecular docking analysis demonstrated that TnBP/DBuP exhibits moderate binding affinity with four core targets: PTGS2, PPARG, APP, and EGFR. In terms of potential mechanisms, TnBP/DBuP is proposed to initiate a self-amplifying neuroinflammatory cascade by inhibiting PPARG, subsequently activating the SOS/RAS/MAPK signaling pathway, thereby exacerbating oxidative stress and neuroinflammation. Additionally, TnBP/DBuP may directly disrupt APP and MMP9, impairing synaptic formation and enhancing cellular damage. These converging pathways damage neural network function, possibly contributing to trouble sleeping. Future studies should focus on validating these mechanistic pathways in real-world exposure settings and further assessing the public health implications of OPFR-induced neurobehavioral effects.

## Figures and Tables

**Table 1:** Baseline characteristics of the control group and the trouble sleeping group.

Characteristic	Control (*N* = 3351)	Trouble Sleeping (*N* = 1234)	*p*-Value
	*N* (%) or Mean ± SD	*N* (%) or Mean ± SD	
Gender			<0.001
Male	1741 (51.95)	514 (41.65)	
Female	1610 (48.05)	720 (58.35)	
Age			<0.001
18–49	1912 (57.06)	507 (41.09)	
50–79	1270 (37.90)	640 (51.86)	
≥80	169 (5.04)	87 (7.05)	
Race			<0.001
Mexican American	554 (16.53)	144 (11.67)	
Other Hispanic	339 (10.12)	119 (9.64)	
Non-Hispanic White	1185 (35.36)	596 (48.30)	
Non-Hispanic Black	706 (21.07)	241 (19.53)	
Non-Hispanic Asian	438 (13.07)	68 (5.51)	
Other Race	129 (3.85)	66 (5.35)	
Asthma			<0.001
Yes	423 (12.62)	268 (21.72)	
No	2928 (87.38)	966 (78.28)	
Smoke status			<0.001
Yes	1282 (38.26)	647 (52.43)	
No	2069 (61.74)	587 (47.57)	
Vigorous activity			<0.001
Yes	890 (26.56)	245 (19.85)	
No	2461 (73.44)	989 (80.15)	
Hypertension			<0.001
Yes	1008 (30.08)	631 (51.13)	
No	2343 (69.92)	603 (48.87)	
Survey cycle			0.5124
2013–2014	1139 (33.99)	420 (34.04)	
2015–2016	994 (29.66)	385 (31.20)	
2017–2018	1218 (36.35)	429 (34.76)	
Body Mass Index (kg/m^2^)	28.87 ± 6.93	31.07 ± 8.18	< 0.001
Depression score	2.42 ±3.41	5.52 ±5.10	<0.001
Sedentary time (min)	363.41 ± 198.99	395.93 ± 211.70	<0.001

## Data Availability

The original contributions presented in this study are included in the article/supplementary material. Further inquiries can be directed to the corresponding authors.

## Supplementary Materials

The following supporting information can be downloaded at: https://www.mdpi.com/article/10.3390/ijms27041934/s1.

## Abbreviations

The following abbreviations are used in this manuscript:

OPFRs	Organophosphate Flame Retardants
POPs	Persistent Organic Pollutants
NHANES	National Health and Nutrition Examination Survey
RCS	Restricted Cubic Splines
WQS	Weighted Quantile Sum
AOP	Adverse Outcome Pathway
MIEs	Molecular Initiating Events
KEs	Key Events
AOs	Adverse Outcomes
KERs	Key Event Relationships
EGFR	Epidermal Growth Factor Receptor
PGE2	Prostaglandin E2
NF-κB	Nuclear Factor-κB
TNF-α	Tumor Necrosis Factor-α
IL-6	Interleukin-6
SHC1	Src Homology and Collagen1
GRB2	Growth Factor Receptor-Bound Protein 2
SOS	Son of Sevenless
RAS	Renin–Angiotensin System
MAPK	Mitogen-Activated Protein Kinase
NRF2	Nuclear Factor Erythroid 2-Related Factor 2
Aβ	Amyloid-β
