# Supplementary material for: Association Between Organophosphate Flame Retardant Exposure and Trouble Sleeping: Integrating Epidemiological Evidence with Mechanistic Insights
Source: Int J Mol Sci. 2026 Feb 18;27(4):1934. doi: 10.3390/ijms27041934 (PMC12940232; doi:10.3390/ijms27041934)
Supplement: Supplementary file 1 [file ijms-27-01934-s001.zip › ijms-4102122-supplementary.pdf]

Supplementary Materials

Association Between Organophosphate Flame Retardant Exposure and Trouble Sleeping: Integrating Epidemiological Evidence with Mechanistic Insights

Yifei Guo [1], Ke Fan [1], Wenhan Tang [1], Caoyue Wu [1], Xin Ni [1], Tianqi Ling [1], Linhao Zong [1,*], Fei Ma [1,2] and Miao Guan [1,2,*]

[1] Jiangsu Key Laboratory for Biodiversity and Biotechnology, College of Life Sciences, Nanjing Normal University, 1 Wenyuan Rd., Nanjing 210023, China; gyf050221@163.com (Y.G.); fk1667298993@163.com (K.F.); 15168706087@163.com (W.T.); 09240314@njnu.edu.cn (C.W.); xin_ni_nnu@163.com (X.N.); 09220235@njnu.edu.cn (T.L.); mafei01@tsinghua.org.cn (F.M.)

[2] Ministry of Education Key Laboratory of NSLSCS, Nanjing Normal University, 1 Wenyuan Rd., Nanjing 210023, China

* Correspondence: zonglinhao@nnu.edu.cn (L.Z.); 08326@njnu.edu.cn (M.G.)

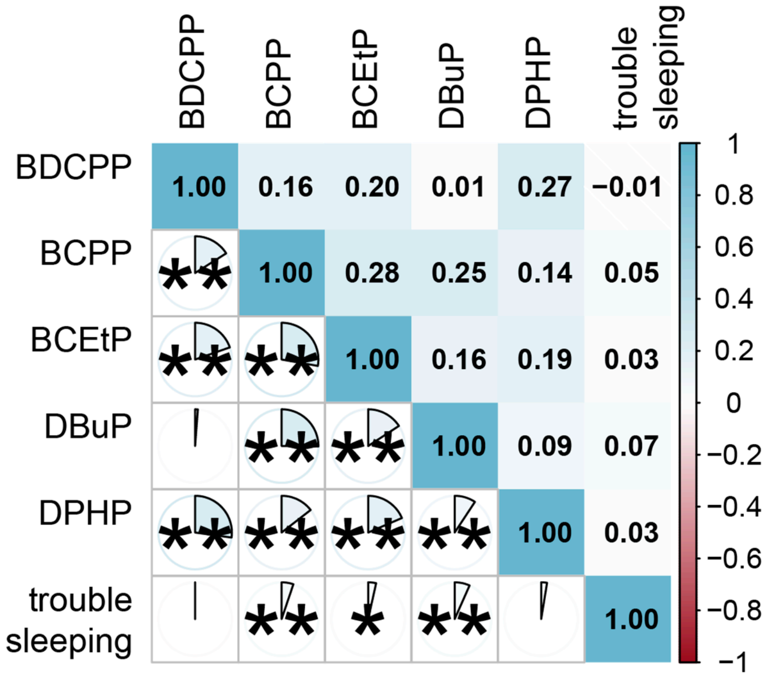

Figure S1. Spearman correlation between BDCPP, BCPP, BCEtP, DBuP, DPHP, and trouble sleeping. Asterisks denote statistical significance: * for $p < 0.05$, and ** for $p < 0.01$.

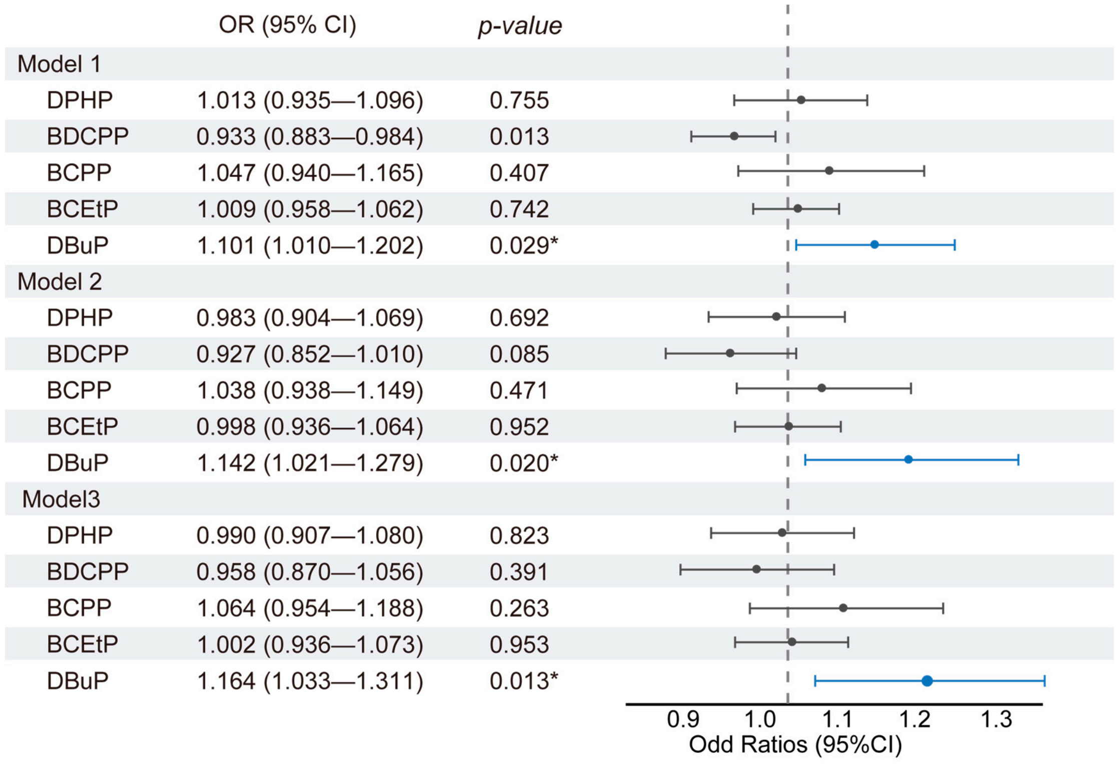

Figure S2. Regression analysis with uncorrected OPFR metabolite concentrations with urinary creatinine as a covariate (sensitivity analysis). Asterisks denote statistical significance: * for $p < 0.05$.

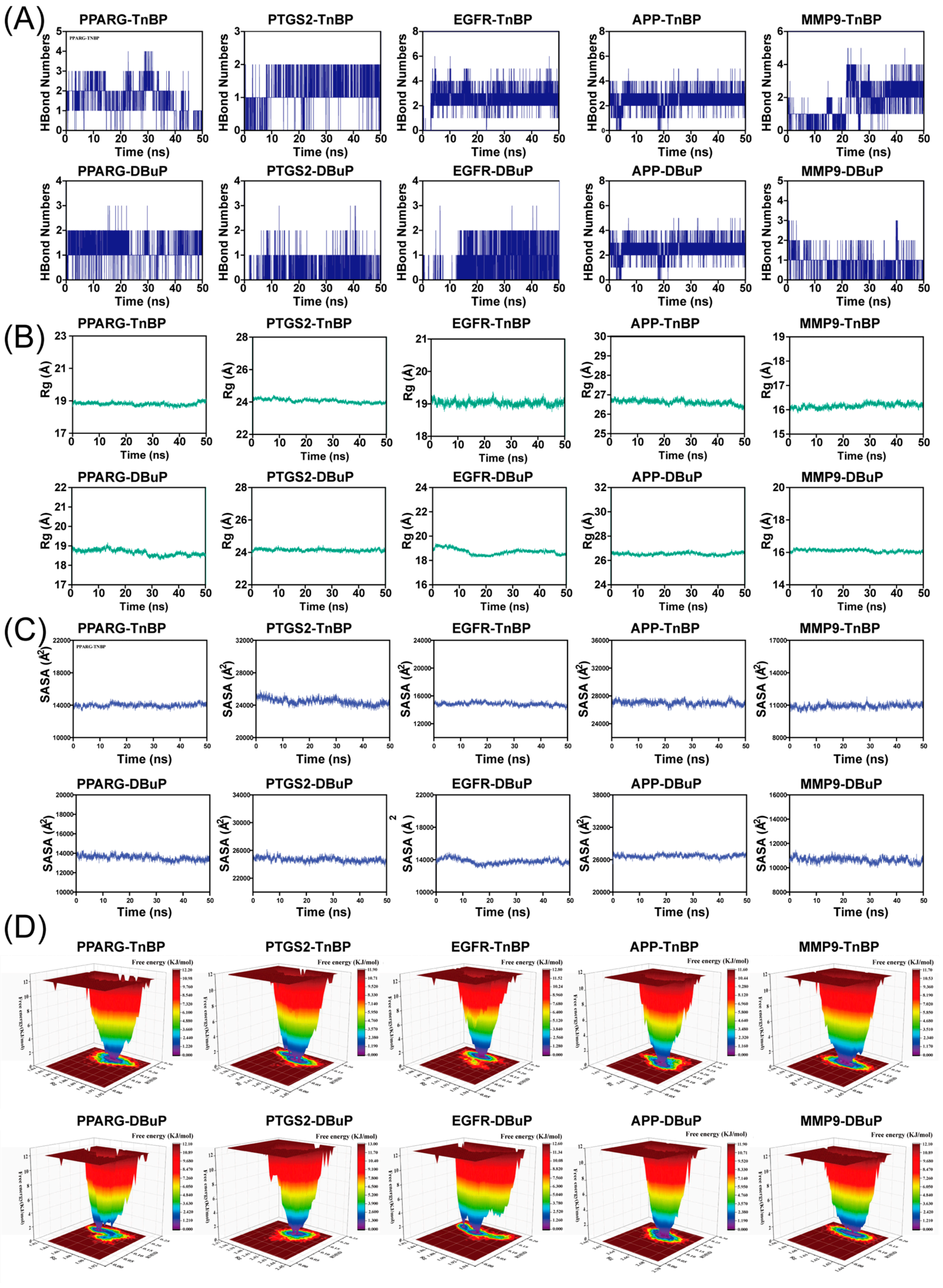

Figure S3. Molecular dynamics simulation of TnBP and DBuP with key target proteins. (**A**) Numbers of hydrogen bonds. (**B**) radius of gyration (Rg), (**C**) solvent accessible surface area (SASA), and (**D**) free energy landscape (FEL) profiles derived from molecular dynamics simulation analysis.

Table S1. NHANES files and variables used in this study.

variable names (NHANES variable codes)	NHANES file names		
	2013–2014	2015–2016	2017–2018
Urinary OPFR metabolite concentrations	Flame Retardant Metabolites - Urine (Surplus)	Flame Retardants - Urine (Surplus)	Flame Retardants - Urine
Trouble sleeping (SLQ050)	Sleep Disorders		
Age (RIDAGEYR)	Demographic Variables and Sample Weights		
Gender (RIAGENDR)	Demographic Variables and Sample Weights		
Race (RIDRETH3)	Demographic Variables and Sample Weights		
Smoking status (SMQ020)	Smoking - Cigarette Use		
Vigorous recreational activity (PAQ650)	Physical Activity		
Hypertension (BPQ020)	Blood Pressure & Cholesterol		
Asthma (MCQ010)	Medical Conditions		
Depression score (DPQ010 - DPQ090)	Mental Health - Depression Screener		
Body Mass Index (BMXBMI)	Body Measures		
Sedentary time (PAD680)	Physical Activity		
Urinary creatinine (URXUCR)	Albumin & Creatinine - Urine		

Table S2. Hub genes identified via three different plugins.

Plugins	Algorithms	Hub Gene Symbols
cytoHubba	Betweenness	*PTGS2, NOS1, ACE, MAOB, SIRT1, ADORA2A, MMP9, EGFR, MAOA, CNR1, ACHE, APP, ESR1, PPARG, DNMT1*
	Closeness	*PTGS2, SLC6A4, COMT, AR, ACE, MAOB, SIRT1, MMP9, EGFR, MAOA, MMP2, ACHE, APP, ESR1, PPARG*
	Degree	*PTGS2, SLC6A4, SLC6A3, COMT, ACE, MAOB, SIRT1, MMP9, EGFR, MAOA, MMP2, HTR2A, APP, ESR1, PPARG*
	EPC	*PTGS2, SLC6A3, COMT, CYP2D6, ACE, MAOB, SIRT1, MMP9, EGFR, MAOA, MMP2, ACHE, APP, ESR1, PPARG*
	MNC	*PTGS2, SLC6A4, SLC6A3, COMT, ACE, MAOB, SIRT1, MMP9, EGFR, MAOA, MMP2, ACHE, APP, ESR1, PPARG*
	Radiality	*PTGS2, COMT, AR, ACE, MAOB, SIRT1, MMP9, EGFR, MAOA, MMP2, CNR1, ACHE, APP, ESR1, PPARG*
	Stress	*PTGS2, COMT, ACE, MAOB, SIRT1, MMP9, EGFR, MAOA, CNR1, ACHE, HTR2A, APP, ESR1, PPARG, DNMT1*
CytoNCA		*ESR1, PPARG, PTGS2, SIRT1, ACE, EGFR, MAOA, MMP9, APP, MAOB, COMT, ACHE, AR, SLC6A4, DNMT1*
MCODE		*EGFR, NOS3, HDAC4, APP, MMP2, PTGS2, MMP9, PPARG, RARB, NOS1, RXRB, SLC6A2, MMP1, TERT, RARA*

Table S3. Significant enrichment of GO BP terms among the five hub genes associated with TnBP/DBuP-induced trouble sleeping ($p < 0.05$).

	Description	Fold Enrichment	p.adjust	Hub Gene Symbols
1	regulation of miRNA transcription	150.880000	0.000181	*APP/EGFR/PPARG*
2	miRNA transcription	148.894737	0.000181	*APP/EGFR/PPARG*
3	regulation of miRNA metabolic process	127.146067	0.000181	*APP/EGFR/PPARG*
4	regulation of apoptotic signaling pathway	37.162562	0.000181	*APP/PTGS2/PPARG/MMP9*
5	neuroinflammatory response	124.351648	0.000181	*APP/PTGS2/MMP9*
6	regulation of inflammatory response	32.800000	0.000217	*APP/PTGS2/PPARG/MMP9*
7	miRNA metabolic process	104.777778	0.000217	*APP/EGFR/PPARG*
8	response to UV-A	502.933333	0.000551	*EGFR/MMP9*
9	placenta development	70.725000	0.000551	*EGFR/PTGS2/PPARG*
10	positive regulation of small molecule metabolic process	67.760479	0.000564	*APP/PTGS2/PPARG*
11	regulation of intrinsic apoptotic signaling pathway	58.030769	0.000817	*APP/PTGS2/MMP9*
12	negative regulation of miRNA transcription	279.407407	0.001375	*APP/PPARG*
13	negative regulation of miRNA metabolic process	260.137931	0.001467	*APP/PPARG*
14	myeloid leukocyte differentiation	44.031128	0.001467	*APP/PPARG/MMP9*
15	cellular response to low-density lipoprotein particle stimulus	193.435897	0.001798	*APP/PPARG*
16	response to lipoprotein particle	188.600000	0.001798	*APP/PPARG*
17	positive regulation of phosphorus metabolic process	35.362500	0.001798	*APP/EGFR/MMP9*
18	positive regulation of phosphate metabolic process	35.362500	0.001798	*APP/EGFR/MMP9*
19	positive regulation of nitric oxide biosynthetic process	179.619048	0.001798	*APP/PTGS2*
20	positive regulation of fatty acid metabolic process	179.619048	0.001798	*PTGS2/PPARG*
21	extracellular matrix organization	34.187311	0.001798	*APP/PPARG/MMP9*
22	extracellular structure organization	34.084337	0.001798	*APP/PPARG/MMP9*
23	external encapsulating structure organization	33.981982	0.001798	*APP/PPARG/MMP9*
24	intrinsic apoptotic signaling pathway	33.779104	0.001798	*APP/PTGS2/MMP9*
25	positive regulation of nitric oxide metabolic process	167.644444	0.001798	*APP/PTGS2*
26	response to light stimulus	33.184751	0.001798	*APP/EGFR/MMP9*
27	regulation of neuroinflammatory response	164.000000	0.001798	*PTGS2/MMP9*
28	cellular response to lipoprotein particle stimulus	160.510638	0.001811	*APP/PPARG*
29	regulation of small molecule metabolic process	31.433333	0.001937	*APP/PTGS2/PPARG*
30	positive regulation of miRNA transcription	134.714286	0.002405	*EGFR/PPARG*
31	response to amyloid-beta	123.672131	0.002764	*APP/MMP9*
32	regulation of nitric oxide biosynthetic process	116.061538	0.002950	*APP/PTGS2*

	Description	Fold Enrichment	p.adjust	Hub Gene Symbols
33	positive regulation of miRNA metabolic process	116.061538	0.002950	*EGFR/PPARG*
34	regulation of nitric oxide metabolic process	109.333333	0.003065	*APP/PTGS2*
35	positive regulation of protein localization	24.653595	0.003065	*APP/EGFR/PPARG*
36	embryo implantation	106.253521	0.003065	*PTGS2/MMP9*
37	response to radiation	24.493506	0.003065	*APP/EGFR/MMP9*
38	regulation of epidermal growth factor receptor signaling pathway	104.777778	0.003065	*EGFR/MMP9*
39	positive regulation of fat cell differentiation	104.777778	0.003065	*PTGS2/PPARG*
40	macrophage differentiation	103.342466	0.003073	*APP/MMP9*
41	myeloid cell differentiation	23.525988	0.003243	*APP/PPARG/MMP9*
42	regulation of ERBB signaling pathway	96.717949	0.003342	*EGFR/MMP9*
43	nitric oxide biosynthetic process	94.300000	0.003434	*APP/PTGS2*
44	nitric oxide metabolic process	84.764045	0.004155	*APP/PTGS2*
45	reactive nitrogen species metabolic process	82.901099	0.004248	*APP/PTGS2*
46	regulation of vascular associated smooth muscle cell proliferation	79.410526	0.004529	*PPARG/MMP9*
47	vascular associated smooth muscle cell proliferation	77.773196	0.004621	*PPARG/MMP9*
48	regulation of fatty acid metabolic process	65.600000	0.006228	*PTGS2/PPARG*
49	negative regulation of intrinsic apoptotic signaling pathway	65.600000	0.006228	*PTGS2/MMP9*
50	fatty acid transport	63.394958	0.006515	*PTGS2/PPARG*
51	regulation of mitochondrion organization	62.866667	0.006515	*PPARG/MMP9*
52	epidermal growth factor receptor signaling pathway	59.401575	0.007155	*EGFR/MMP9*
53	positive regulation of mitotic cell cycle	54.666667	0.008284	*APP/EGFR*
54	ERBB signaling pathway	52.027586	0.008810	*EGFR/MMP9*
55	positive regulation of lipid metabolic process	52.027586	0.008810	*PTGS2/PPARG*
56	regulation of fat cell differentiation	50.972973	0.008974	*PTGS2/PPARG*
57	regulation of ketone metabolic process	50.630872	0.008974	*PTGS2/PPARG*
58	positive regulation of apoptotic signaling pathway	48.987013	0.009418	*APP/PPARG*
59	response to UV	47.446541	0.009542	*EGFR/MMP9*
60	regulation of blood vessel endothelial cell migration	47.446541	0.009542	*PTGS2/PPARG*
61	cellular response to hypoxia	47.446541	0.009542	*PTGS2/PPARG*
62	cellular response to decreased oxygen levels	45.173653	0.010351	*PTGS2/PPARG*
63	regulation of smooth muscle cell proliferation	43.860465	0.010802	*PPARG/MMP9*
64	smooth muscle cell proliferation	42.863636	0.011131	*PPARG/MMP9*
65	positive regulation of inflammatory response	42.145251	0.011286	*APP/PTGS2*
66	monocarboxylic acid transport	41.911111	0.011286	*PTGS2/PPARG*
67	cellular response to oxygen levels	41.224044	0.011489	*PTGS2/PPARG*
68	blood vessel endothelial cell migration	40.342246	0.011816	*PTGS2/PPARG*

	Description	Fold Enrichment	p.adjust	Hub Gene Symbols
69	female pregnancy	38.294416	0.012914	*PTGS2/MMP9*
70	positive regulation of ERK1 and ERK2 cascade	37.532338	0.013247	*APP/EGFR*
71	regulation of blood pressure	37.162562	0.013320	*PTGS2/PPARG*
72	multi-organism reproductive process	34.764977	0.014786	*PTGS2/MMP9*
73	positive regulation of phosphatidylinositol 3-kinase/protein kinase B signal transduction	34.764977	0.014786	*APP/EGFR*
74	cell maturation	34.135747	0.015123	*APP/PPARG*
75	multi-multicellular organism process	33.233480	0.015735	*PTGS2/MMP9*
76	positive regulation of protein phosphorylation	32.658009	0.016074	*EGFR/MMP9*
77	ketone metabolic process	31.831224	0.016691	*PTGS2/PPARG*
78	regulation of endothelial cell migration	31.564854	0.016754	*PTGS2/PPARG*
79	negative regulation of apoptotic signaling pathway	30.791837	0.017373	*PTGS2/MMP9*
80	muscle cell proliferation	29.936508	0.018139	*PPARG/MMP9*
81	positive regulation of phosphorylation	29.700787	0.018197	*EGFR/MMP9*
82	fat cell differentiation	29.127413	0.018681	*PTGS2/PPARG*
83	positive regulation of protein transport	28.684411	0.019024	*APP/PPARG*
84	regulation of canonical Wnt signaling pathway	27.136691	0.020973	*APP/EGFR*
85	learning or memory	26.563380	0.021619	*APP/EGFR*
86	anatomical structure maturation	26.285714	0.021815	*APP/PPARG*
87	endothelial cell migration	24.980132	0.022473	*PTGS2/PPARG*
88	regulation of ERK1 and ERK2 cascade	24.897690	0.022473	*APP/EGFR*
89	regulation of phosphatidylinositol 3-kinase/protein kinase B signal transduction	24.257235	0.022473	*APP/EGFR*
90	fever generation	377.200000	0.022473	*PTGS2*
91	wound healing involved in inflammatory response	377.200000	0.022473	*PPARG*
92	intrinsic apoptotic signaling pathway in response to osmotic stress	377.200000	0.022473	*PTGS2*
93	cellular response to manganese ion	377.200000	0.022473	*APP*
94	fibroblast growth factor production	377.200000	0.022473	*PTGS2*
95	regulation of fibroblast growth factor production	377.200000	0.022473	*PTGS2*
96	response to hypoxia	23.798107	0.022473	*PTGS2/PPARG*
97	response to selenium ion	342.909091	0.022473	*PTGS2*
98	negative regulation of cardiac muscle adaptation	342.909091	0.022473	*PPARG*
99	negative regulation of cholesterol storage	342.909091	0.022473	*PPARG*
100	lipoxygenase pathway	342.909091	0.022473	*PTGS2*
101	positive regulation of heat generation	342.909091	0.022473	*PTGS2*
102	cellular response to UV-A	342.909091	0.022473	*MMP9*
103	negative regulation of cardiac muscle hypertrophy in response to stress	342.909091	0.022473	*PPARG*
104	positive regulation of vascular associated smooth muscle cell apoptotic process	342.909091	0.022473	*PPARG*

	Description	Fold Enrichment	p.adjust	Hub Gene Symbols
105	positive regulation of unsaturated fatty acid biosynthetic process	342.909091	0.022473	*PTGS2*
106	response to decreased oxygen levels	22.860606	0.022473	*PTGS2/PPARG*
107	ERK1 and ERK2 cascade	22.791541	0.022473	*APP/EGFR*
108	cognition	22.654655	0.022473	*APP/EGFR*
109	canonical Wnt signaling pathway	22.654655	0.022473	*APP/EGFR*
110	positive regulation of establishment of protein localization	22.586826	0.022473	*APP/PPARG*
111	protein import	314.333333	0.022473	*APP*
112	antifungal humoral response	314.333333	0.022473	*APP*
113	positive regulation of keratinocyte migration	314.333333	0.022473	*MMP9*
114	negative regulation of extracellular matrix assembly	314.333333	0.022473	*PPARG*
115	positive regulation of cell cycle	21.616046	0.022473	*APP/EGFR*
116	phosphatidylinositol 3-kinase/protein kinase B signal transduction	21.310734	0.022473	*APP/EGFR*
117	cellular response to abiotic stimulus	21.250704	0.022473	*PTGS2/MMP9*
118	cellular response to environmental stimulus	21.250704	0.022473	*PTGS2/MMP9*
119	regulation of Wnt signaling pathway	21.191011	0.022473	*APP/EGFR*
120	carboxylic acid transport	21.191011	0.022473	*PTGS2/PPARG*
121	organic acid transport	21.131653	0.022473	*PTGS2/PPARG*
122	response to oxygen levels	21.131653	0.022473	*PTGS2/PPARG*
123	Toll signaling pathway	290.153846	0.022473	*APP*
124	spontaneous synaptic transmission	290.153846	0.022473	*APP*
125	regulation of cellular response to osmotic stress	290.153846	0.022473	*PTGS2*
126	negative regulation of long-term synaptic potentiation	290.153846	0.022473	*APP*
127	positive regulation of adipose tissue development	290.153846	0.022473	*PPARG*
128	neuron projection maintenance	290.153846	0.022473	*APP*
129	regulation of lipid metabolic process	21.072626	0.022473	*PTGS2/PPARG*
130	developmental maturation	20.725275	0.022483	*APP/PPARG*
131	negative regulation of macrophage derived foam cell differentiation	269.428571	0.022483	*PPARG*
132	regulation of heat generation	269.428571	0.022483	*PTGS2*
133	regulation of keratinocyte migration	269.428571	0.022483	*MMP9*
134	eyelid development in camera-type eye	269.428571	0.022483	*EGFR*
135	negative regulation of cellular response to transforming growth factor beta stimulus	269.428571	0.022483	*PPARG*
136	negative regulation of cardiocyte differentiation	269.428571	0.022483	*EGFR*
137	positive regulation of protein modification process	20.117333	0.022483	*EGFR/MMP9*
138	regulation of protein catabolic process	19.697128	0.022483	*APP/EGFR*
139	response to yeast	251.466667	0.022483	*APP*
140	response to manganese ion	251.466667	0.022483	*APP*
141	negative regulation of muscle adaptation	251.466667	0.022483	*PPARG*

	Description	Fold Enrichment	p.adjust	Hub Gene Symbols
142	neuron remodeling	251.466667	0.022483	*APP*
143	regulation of prostaglandin biosynthetic process	251.466667	0.022483	*PTGS2*
144	regulation of response to osmotic stress	251.466667	0.022483	*PTGS2*
145	regulation of response to type II interferon	251.466667	0.022483	*PPARG*
146	regulation of type II interferon-mediated signaling pathway	251.466667	0.022483	*PPARG*
147	regulation of dendritic spine maintenance	251.466667	0.022483	*APP*
148	regulation of connective tissue replacement	251.466667	0.022483	*PPARG*
149	regulation of protein phosphorylation	19.493540	0.022671	*EGFR/MMP9*
150	microglia differentiation	235.750000	0.023196	*APP*
151	positive regulation of brown fat cell differentiation	235.750000	0.023196	*PTGS2*
152	positive regulation of cAMP/PKA signal transduction	235.750000	0.023196	*APP*
153	regulation of epithelial cell differentiation involved in kidney development	235.750000	0.023196	*MMP9*
154	intracellular copper ion homeostasis	221.882353	0.024015	*APP*
155	prostaglandin secretion	221.882353	0.024015	*PTGS2*
156	positive regulation of membrane protein ectodomain proteolysis	221.882353	0.024015	*APP*
157	regulation of unsaturated fatty acid biosynthetic process	221.882353	0.024015	*PTGS2*
158	regulation of epithelial cell proliferation	18.134615	0.024192	*EGFR/PPARG*
159	mitochondrion organization	18.091127	0.024192	*PPARG/MMP9*
160	fatty acid metabolic process	17.919240	0.024192	*PTGS2/PPARG*
161	G protein-coupled receptor internalization	209.555556	0.024192	*APP*
162	protein trimerization	209.555556	0.024192	*APP*
163	vascular associated smooth muscle cell apoptotic process	209.555556	0.024192	*PPARG*
164	regulation of vascular associated smooth muscle cell apoptotic process	209.555556	0.024192	*PPARG*
165	regulation of amyloid fibril formation	209.555556	0.024192	*APP*
166	regulation of phosphorylation	17.503480	0.024638	*EGFR/MMP9*
167	response to lead ion	198.526316	0.024638	*APP*
168	regulation of cholesterol storage	198.526316	0.024638	*PPARG*
169	heat generation	198.526316	0.024638	*PTGS2*
170	positive regulation of smooth muscle cell apoptotic process	198.526316	0.024638	*PPARG*
171	white fat cell differentiation	198.526316	0.024638	*PPARG*
172	positive regulation of superoxide anion generation	188.600000	0.025195	*APP*
173	keratinocyte migration	188.600000	0.025195	*MMP9*
174	regulation of neurotransmitter uptake	188.600000	0.025195	*APP*
175	positive regulation of transforming growth factor beta production	188.600000	0.025195	*PTGS2*
176	regulation of adipose tissue development	188.600000	0.025195	*PPARG*
177	ossification	16.580220	0.025721	*EGFR/PPARG*

	Description	Fold Enrichment	p.adjust	Hub Gene Symbols
178	regulation of cardiac muscle adaptation	179.619048	0.025721	*PPARG*
179	copper ion homeostasis	179.619048	0.025721	*APP*
180	regulation of amyloid-beta clearance	179.619048	0.025721	*APP*
181	regulation of cardiac muscle hypertrophy in response to stress	179.619048	0.025721	*PPARG*
182	regulation of protein transport	16.471616	0.025808	*APP/PPARG*
183	lipid transport	16.364425	0.025940	*PTGS2/PPARG*
184	organic anion transport	16.188841	0.025940	*PTGS2/PPARG*
185	desensitization of G protein-coupled receptor signaling pathway	171.454545	0.025940	*APP*
186	regulation of superoxide anion generation	171.454545	0.025940	*APP*
187	positive regulation of peptidyl-serine phosphorylation	171.454545	0.025940	*EGFR*
188	negative regulation of tissue remodeling	171.454545	0.025940	*PPARG*
189	positive regulation of MAPK cascade	15.915612	0.026143	*APP/EGFR*
190	negative regulation of lipid storage	164.000000	0.026143	*PPARG*
191	negative adaptation of signaling pathway	164.000000	0.026143	*APP*
192	adaptation of signaling pathway	164.000000	0.026143	*APP*
193	ERBB2 signaling pathway	164.000000	0.026143	*EGFR*
194	positive regulation of DNA binding	164.000000	0.026143	*MMP9*
195	positive regulation of monocyte chemotaxis	164.000000	0.026143	*APP*
196	lipid localization	15.619048	0.026190	*PTGS2/PPARG*
197	Wnt signaling pathway	15.490760	0.026190	*APP/EGFR*
198	positive regulation of nucleotide catabolic process	157.166667	0.026190	*APP*
199	positive regulation of purine nucleotide catabolic process	157.166667	0.026190	*APP*
200	positive regulation of glycolytic process	157.166667	0.026190	*APP*
201	dendritic spine maintenance	157.166667	0.026190	*APP*
202	positive regulation of long-term synaptic potentiation	157.166667	0.026190	*APP*
203	epithelial cell proliferation	15.209677	0.026190	*EGFR/PPARG*
204	cholesterol storage	150.880000	0.026190	*PPARG*
205	decidualization	150.880000	0.026190	*PTGS2*
206	morphogenesis of an epithelial fold	150.880000	0.026190	*EGFR*
207	positive regulation of release of cytochrome c from mitochondria	150.880000	0.026190	*MMP9*
208	regulation of brown fat cell differentiation	150.880000	0.026190	*PTGS2*
209	connective tissue replacement	150.880000	0.026190	*PPARG*
210	regulation of extracellular matrix assembly	150.880000	0.026190	*PPARG*
211	negative regulation of vascular endothelial cell proliferation	150.880000	0.026190	*PPARG*
212	cell growth	15.118236	0.026190	*APP/EGFR*
213	lipoprotein transport	145.076923	0.026426	*PPARG*
214	positive regulation of fatty acid biosynthetic process	145.076923	0.026426	*PTGS2*

	Description	Fold Enrichment	p.adjust	Hub Gene Symbols
215	astrocyte activation	145.076923	0.026426	*APP*
216	regulation of long-term neuronal synaptic plasticity	145.076923	0.026426	*APP*
217	cellular response to copper ion	145.076923	0.026426	*APP*
218	negative regulation of blood circulation	145.076923	0.026426	*APP*
219	positive regulation of cholesterol efflux	139.703704	0.026945	*PPARG*
220	lipoprotein localization	139.703704	0.026945	*PPARG*
221	positive regulation of amyloid precursor protein catabolic process	139.703704	0.026945	*APP*
222	negative regulation of extracellular matrix organization	139.703704	0.026945	*PPARG*
223	positive regulation of acute inflammatory response	134.714286	0.027568	*PTGS2*
224	prostaglandin transport	134.714286	0.027568	*PTGS2*
225	regulation of membrane protein ectodomain proteolysis	134.714286	0.027568	*APP*
226	positive regulation of receptor internalization	130.068966	0.028298	*APP*
227	type II interferon-mediated signaling pathway	130.068966	0.028298	*PPARG*
228	peroxisome proliferator activated receptor signaling pathway	125.733333	0.028889	*PPARG*
229	negative regulation of epidermal growth factor receptor signaling pathway	125.733333	0.028889	*EGFR*
230	inflammatory response to wounding	125.733333	0.028889	*PPARG*
231	prostaglandin biosynthetic process	121.677419	0.029338	*PTGS2*
232	regulation of peptidyl-serine phosphorylation	121.677419	0.029338	*EGFR*
233	ionotropic glutamate receptor signaling pathway	121.677419	0.029338	*APP*
234	regulation of mitochondrial fission	121.677419	0.029338	*PPARG*
235	positive regulation of vascular endothelial growth factor production	117.875000	0.029525	*PTGS2*
236	positive regulation of epidermal growth factor receptor signaling pathway	117.875000	0.029525	*MMP9*
237	collateral sprouting	117.875000	0.029525	*APP*
238	regulation of monocyte chemotaxis	117.875000	0.029525	*APP*
239	regulation of superoxide metabolic process	117.875000	0.029525	*APP*
240	regulation of presynapse assembly	117.875000	0.029525	*APP*
241	salivary gland morphogenesis	114.303030	0.029823	*EGFR*
242	prostanoid biosynthetic process	114.303030	0.029823	*PTGS2*
243	regulation of presynapse organization	114.303030	0.029823	*APP*
244	regulation of cardiocyte differentiation	114.303030	0.029823	*EGFR*
245	positive regulation of excitatory postsynaptic potential	114.303030	0.029823	*APP*
246	cellular response to fluid shear stress	110.941176	0.030351	*PTGS2*
247	negative regulation of ERBB signaling pathway	110.941176	0.030351	*EGFR*
248	positive regulation of ERBB signaling pathway	110.941176	0.030351	*MMP9*

	Description	Fold Enrichment	p.adjust	Hub Gene Symbols
249	muscle hypertrophy in response to stress	107.771429	0.030503	*PPARG*
250	positive regulation of muscle cell apoptotic process	107.771429	0.030503	*PPARG*
251	regulation of macrophage derived foam cell differentiation	107.771429	0.030503	*PPARG*
252	cardiac muscle adaptation	107.771429	0.030503	*PPARG*
253	cardiac muscle hypertrophy in response to stress	107.771429	0.030503	*PPARG*
254	regulation of transforming growth factor beta production	107.771429	0.030503	*PTGS2*
255	maternal placenta development	104.777778	0.030530	*PTGS2*
256	mating behavior	104.777778	0.030530	*APP*
257	positive regulation of sterol transport	104.777778	0.030530	*PPARG*
258	positive regulation of cholesterol transport	104.777778	0.030530	*PPARG*
259	cellular response to estradiol stimulus	104.777778	0.030530	*EGFR*
260	regulation of endoplasmic reticulum stress-induced intrinsic apoptotic signaling pathway	104.777778	0.030530	*APP*
261	negative regulation of receptor signaling pathway via STAT	104.777778	0.030530	*PPARG*
262	salivary gland development	101.945946	0.031018	*EGFR*
263	positive regulation of cell migration involved in sprouting angiogenesis	101.945946	0.031018	*PTGS2*
264	positive regulation of T cell migration	101.945946	0.031018	*APP*
265	monocyte differentiation	99.263158	0.031733	*PPARG*
266	regulation of smooth muscle cell apoptotic process	96.717949	0.032080	*PPARG*
267	retinoic acid receptor signaling pathway	96.717949	0.032080	*PPARG*
268	transforming growth factor beta production	96.717949	0.032080	*PTGS2*
269	positive regulation of ATP metabolic process	96.717949	0.032080	*APP*
270	negative regulation of cardiac muscle hypertrophy	94.300000	0.032299	*PPARG*
271	macrophage derived foam cell differentiation	94.300000	0.032299	*PPARG*
272	smooth muscle cell apoptotic process	94.300000	0.032299	*PPARG*
273	long-chain fatty acid biosynthetic process	94.300000	0.032299	*PTGS2*
274	response to copper ion	94.300000	0.032299	*APP*
275	regulation of toll-like receptor signaling pathway	92.000000	0.032744	*APP*
276	foam cell differentiation	92.000000	0.032744	*PPARG*
277	positive regulation of response to endoplasmic reticulum stress	92.000000	0.032744	*APP*
278	negative regulation of muscle hypertrophy	89.809524	0.032828	*PPARG*
279	positive regulation of DNA replication	89.809524	0.032828	*EGFR*
280	positive regulation of nucleotide metabolic process	89.809524	0.032828	*APP*

	Description	Fold Enrichment	p.adjust	Hub Gene Symbols
281	negative regulation of SMAD protein signal transduction	89.809524	0.032828	*PPARG*
282	amyloid-beta clearance	89.809524	0.032828	*APP*
283	positive regulation of purine nucleotide metabolic process	89.809524	0.032828	*APP*
284	mitochondrial fission	87.720930	0.033254	*PPARG*
285	astrocyte development	87.720930	0.033254	*APP*
286	negative regulation of vascular associated smooth muscle cell proliferation	87.720930	0.033254	*PPARG*
287	icosanoid secretion	85.727273	0.033670	*PTGS2*
288	positive regulation of G protein-coupled receptor signaling pathway	85.727273	0.033670	*APP*
289	positive regulation of lymphocyte migration	85.727273	0.033670	*APP*
290	collagen catabolic process	83.822222	0.033732	*MMP9*
291	response to fluid shear stress	83.822222	0.033732	*PTGS2*
292	superoxide anion generation	83.822222	0.033732	*APP*
293	regulation of release of cytochrome c from mitochondria	83.822222	0.033732	*MMP9*
294	regulation of long-term synaptic potentiation	83.822222	0.033732	*APP*
295	amyloid fibril formation	83.822222	0.033732	*APP*
296	presynapse assembly	82.000000	0.034361	*APP*
297	neurotransmitter uptake	80.255319	0.034407	*APP*
298	acute-phase response	80.255319	0.034407	*PTGS2*
299	regulation of DNA binding	80.255319	0.034407	*MMP9*
300	cellular response to epidermal growth factor stimulus	80.255319	0.034407	*EGFR*
301	regulation of cAMP/PKA signal transduction	80.255319	0.034407	*APP*
302	ligand-gated ion channel signaling pathway	80.255319	0.034407	*APP*
303	microglial cell activation	78.583333	0.034676	*APP*
304	neuron recognition	78.583333	0.034676	*APP*
305	mRNA transcription by RNA polymerase II	78.583333	0.034676	*PPARG*
306	negative regulation of protein localization to nucleus	78.583333	0.034676	*APP*
307	regulation of acute inflammatory response	76.979592	0.034825	*PTGS2*
308	membrane protein ectodomain proteolysis	76.979592	0.034825	*APP*
309	regulation of lipid storage	76.979592	0.034825	*PPARG*
310	reproductive behavior	76.979592	0.034825	*APP*
311	positive regulation of binding	76.979592	0.034825	*MMP9*
312	prostaglandin metabolic process	75.440000	0.035081	*PTGS2*
313	positive regulation of epithelial cell migration	75.440000	0.035081	*MMP9*
314	epithelial cell differentiation involved in kidney development	75.440000	0.035081	*MMP9*
315	cellular response to amyloid-beta	75.440000	0.035081	*APP*

	Description	Fold Enrichment	p.adjust	Hub Gene Symbols
316	leukocyte activation involved in inflammatory response	73.960784	0.035165	*APP*
317	exocrine system development	73.960784	0.035165	*EGFR*
318	modulation of excitatory postsynaptic potential	73.960784	0.035165	*APP*
319	cellular response to nerve growth factor stimulus	73.960784	0.035165	*APP*
320	prostanoid metabolic process	72.538462	0.035165	*PTGS2*
321	response to epidermal growth factor	72.538462	0.035165	*EGFR*
322	lipid export from cell	72.538462	0.035165	*PTGS2*
323	regulation of vascular endothelial cell proliferation	72.538462	0.035165	*PPARG*
324	regulation of T cell migration	72.538462	0.035165	*APP*
325	arachidonate metabolic process	71.169811	0.035165	*PTGS2*
326	cerebral cortex cell migration	71.169811	0.035165	*EGFR*
327	endodermal cell differentiation	71.169811	0.035165	*MMP9*
328	ephrin receptor signaling pathway	71.169811	0.035165	*MMP9*
329	digestive tract morphogenesis	71.169811	0.035165	*EGFR*
330	brown fat cell differentiation	71.169811	0.035165	*PTGS2*
331	extracellular matrix assembly	71.169811	0.035165	*PPARG*
332	vascular endothelial cell proliferation	71.169811	0.035165	*PPARG*
333	positive regulation of vascular associated smooth muscle cell proliferation	71.169811	0.035165	*MMP9*
334	monocyte chemotaxis	69.851852	0.035399	*APP*
335	visual learning	69.851852	0.035399	*APP*
336	positive regulation of receptor-mediated endocytosis	69.851852	0.035399	*APP*
337	response to nerve growth factor	69.851852	0.035399	*APP*
338	negative regulation of epithelial cell differentiation	68.581818	0.035838	*MMP9*
339	neuron maturation	68.581818	0.035838	*APP*
340	regulation of glycolytic process	67.357143	0.035852	*APP*
341	regulation of nucleotide catabolic process	67.357143	0.035852	*APP*
342	regulation of purine nucleotide catabolic process	67.357143	0.035852	*APP*
343	glial cell activation	67.357143	0.035852	*APP*
344	cAMP/PKA signal transduction	67.357143	0.035852	*APP*
345	positive regulation of G1/S transition of mitotic cell cycle	67.357143	0.035852	*EGFR*
346	regulation of cholesterol efflux	66.175439	0.036070	*PPARG*
347	striated muscle adaptation	66.175439	0.036070	*PPARG*
348	positive regulation of fibroblast proliferation	66.175439	0.036070	*EGFR*
349	presynapse organization	66.175439	0.036070	*APP*
350	cellular response to cAMP	65.034483	0.036593	*APP*
351	release of cytochrome c from mitochondria	63.932203	0.036593	*MMP9*
352	regulation of fatty acid biosynthetic process	63.932203	0.036593	*PTGS2*

	Description	Fold Enrichment	p.adjust	Hub Gene Symbols
353	positive regulation of calcium-mediated signaling	63.932203	0.036593	*APP*
354	cell differentiation involved in kidney development	63.932203	0.036593	*MMP9*
355	cellular response to osmotic stress	63.932203	0.036593	*PTGS2*
356	positive regulation of extrinsic apoptotic signaling pathway	63.932203	0.036593	*APP*
357	unsaturated fatty acid biosynthetic process	62.866667	0.036899	*PTGS2*
358	maternal process involved in female pregnancy	62.866667	0.036899	*PTGS2*
359	regulation of amyloid precursor protein catabolic process	62.866667	0.036899	*APP*
360	mRNA transcription	61.836066	0.036995	*PPARG*
361	regulation of neuronal synaptic plasticity	61.836066	0.036995	*APP*
362	endocrine hormone secretion	61.836066	0.036995	*PPARG*
363	icosanoid transport	61.836066	0.036995	*PTGS2*
364	positive regulation of gene expression, epigenetic	61.836066	0.036995	*APP*
365	endoderm formation	60.838710	0.037188	*MMP9*
366	visual behavior	60.838710	0.037188	*APP*
367	regulation of vascular endothelial growth factor production	60.838710	0.037188	*PTGS2*
368	cellular response to catecholamine stimulus	60.838710	0.037188	*APP*
369	icosanoid biosynthetic process	59.873016	0.037682	*PTGS2*
370	membrane protein proteolysis	58.937500	0.037967	*APP*
371	cellular response to monoamine stimulus	58.937500	0.037967	*APP*
372	response to catecholamine	58.937500	0.037967	*APP*
373	glutamate receptor signaling pathway	58.030769	0.038248	*APP*
374	vascular endothelial growth factor production	58.030769	0.038248	*PTGS2*
375	long-chain fatty acid transport	58.030769	0.038248	*PPARG*
376	telencephalon cell migration	57.151515	0.038422	*EGFR*
377	positive regulation of interleukin-1 beta production	57.151515	0.038422	*APP*
378	response to monoamine	57.151515	0.038422	*APP*
379	positive regulation of non-canonical NF-kappaB signal transduction	57.151515	0.038422	*APP*
380	intrinsic apoptotic signaling pathway in response to endoplasmic reticulum stress	56.298507	0.038898	*APP*
381	regulation of multicellular organism growth	55.470588	0.039063	*APP*
382	regulation of carbohydrate catabolic process	55.470588	0.039063	*APP*
383	defense response to fungus	55.470588	0.039063	*APP*
384	positive regulation of protein localization to plasma membrane	55.470588	0.039063	*EGFR*
385	forebrain cell migration	54.666667	0.039326	*EGFR*
386	positive regulation of neuron apoptotic process	54.666667	0.039326	*APP*

	Description	Fold Enrichment	p.adjust	Hub Gene Symbols
387	negative regulation of G protein-coupled receptor signaling pathway	54.666667	0.039326	*APP*
388	positive regulation of cell cycle G1/S phase transition	53.885714	0.039686	*EGFR*
389	negative regulation of lipid localization	53.885714	0.039686	*PPARG*
390	negative regulation of osteoblast differentiation	53.126761	0.039738	*PPARG*
391	negative regulation of smooth muscle cell proliferation	53.126761	0.039738	*PPARG*
392	protein insertion into membrane	53.126761	0.039738	*EGFR*
393	adipose tissue development	53.126761	0.039738	*PPARG*
394	positive regulation of reactive oxygen species metabolic process	53.126761	0.039738	*APP*
395	regulation of epithelial cell migration	52.388889	0.039789	*MMP9*
396	peptidyl-serine phosphorylation	52.388889	0.039789	*EGFR*
397	extracellular matrix disassembly	52.388889	0.039789	*MMP9*
398	positive chemotaxis	52.388889	0.039789	*APP*
399	regulation of lymphocyte migration	52.388889	0.039789	*APP*
400	positive regulation of chemokine production	51.671233	0.040236	*APP*
401	regulation of cardiac muscle hypertrophy	50.972973	0.040480	*PPARG*
402	peptidyl-serine modification	50.972973	0.040480	*EGFR*
403	amyloid precursor protein catabolic process	50.972973	0.040480	*APP*
404	superoxide metabolic process	49.631579	0.041360	*APP*
405	regulation of extracellular matrix organization	49.631579	0.041360	*PPARG*
406	regulation of receptor internalization	48.987013	0.041388	*APP*
407	regulation of muscle hypertrophy	48.987013	0.041388	*PPARG*
408	negative regulation of BMP signaling pathway	48.987013	0.041388	*PPARG*
409	positive regulation of type II interferon production	48.987013	0.041388	*APP*
410	positive regulation of interleukin-1 production	48.987013	0.041388	*APP*
411	negative regulation of endothelial cell proliferation	48.358974	0.041543	*PPARG*
412	antibacterial humoral response	48.358974	0.041543	*APP*
413	response to fungus	47.746835	0.041543	*APP*
414	regulation of sterol transport	47.746835	0.041543	*PPARG*
415	regulation of cholesterol transport	47.746835	0.041543	*PPARG*
416	cholesterol efflux	47.746835	0.041543	*PPARG*
417	regulation of peptidyl-tyrosine phosphorylation	47.746835	0.041543	*EGFR*
418	T cell migration	47.746835	0.041543	*APP*
419	regulation of ATP metabolic process	47.746835	0.041543	*APP*
420	positive regulation of blood vessel endothelial cell migration	47.150000	0.041765	*PTGS2*
421	negative regulation of cation transmembrane transport	47.150000	0.041765	*MMP9*

	Description	Fold Enrichment	p.adjust	Hub Gene Symbols
422	positive regulation of protein localization to cell periphery	47.150000	0.041765	*EGFR*
423	regulation of cellular response to insulin stimulus	46.567901	0.042182	*PPARG*
424	regulation of cell migration involved in sprouting angiogenesis	46.000000	0.042598	*PTGS2*
425	negative regulation of blood vessel endothelial cell migration	45.445783	0.042810	*PPARG*
426	cellular component maintenance	45.445783	0.042810	*APP*
427	dendritic spine organization	45.445783	0.042810	*APP*
428	positive regulation of carbohydrate metabolic process	44.904762	0.043119	*APP*
429	regulation of SMAD protein signal transduction	44.904762	0.043119	*PPARG*
430	axo-dendritic transport	44.376471	0.043125	*APP*
431	positive regulation of lipid transport	44.376471	0.043125	*PPARG*
432	regulation of tissue remodeling	44.376471	0.043125	*PPARG*
433	negative regulation of monoatomic ion transmembrane transport	44.376471	0.043125	*MMP9*
434	lipoprotein metabolic process	44.376471	0.043125	*APP*
435	response to osmotic stress	43.356322	0.043928	*PTGS2*
436	adult locomotory behavior	43.356322	0.043928	*APP*
437	toll-like receptor signaling pathway	42.863636	0.044327	*APP*
438	regulation of nucleotide metabolic process	42.382022	0.044610	*APP*
439	regulation of purine nucleotide metabolic process	42.382022	0.044610	*APP*
440	endoderm development	41.911111	0.044610	*MMP9*
441	astrocyte differentiation	41.911111	0.044610	*APP*
442	response to cAMP	41.911111	0.044610	*APP*
443	cellular oxidant detoxification	41.911111	0.044610	*PTGS2*
444	positive regulation of protein localization to membrane	41.911111	0.044610	*EGFR*
445	embryonic placenta development	41.450549	0.045000	*EGFR*
446	regulation of muscle cell apoptotic process	41.000000	0.045286	*PPARG*
447	cellular response to UV	41.000000	0.045286	*MMP9*
448	cellular response to amino acid stimulus	40.559140	0.045569	*EGFR*
449	neuron projection organization	40.559140	0.045569	*APP*
450	regulation of fibroblast proliferation	40.127660	0.045952	*EGFR*
451	regulation of response to endoplasmic reticulum stress	39.705263	0.046333	*APP*
452	associative learning	39.291667	0.046609	*APP*
453	lipid storage	39.291667	0.046609	*PPARG*
454	positive regulation of leukocyte chemotaxis	38.886598	0.046780	*APP*
455	adenylate cyclase-inhibiting G protein-coupled receptor signaling pathway	38.886598	0.046780	*APP*
456	positive regulation of neuron differentiation	38.886598	0.046780	*APP*

	Description	Fold Enrichment	p.adjust	Hub Gene Symbols
457	positive regulation of smooth muscle cell proliferation	38.489796	0.047012	*MMP9*
458	positive regulation of mononuclear cell migration	38.489796	0.047012	*APP*
459	hair follicle development	38.101010	0.047012	*EGFR*
460	muscle cell apoptotic process	38.101010	0.047012	*PPARG*
461	regulation of muscle adaptation	38.101010	0.047012	*PPARG*
462	regulation of calcium-mediated signaling	38.101010	0.047012	*APP*
463	regulation of receptor signaling pathway via STAT	38.101010	0.047012	*PPARG*
464	protein tetramerization	37.720000	0.047177	*APP*
465	SMAD protein signal transduction	37.720000	0.047177	*PPARG*
466	myeloid cell development	37.720000	0.047177	*APP*
467	nutrient storage	37.346535	0.047198	*PPARG*
468	cell migration involved in sprouting angiogenesis	36.980392	0.047198	*PTGS2*
469	negative regulation of endothelial cell migration	36.980392	0.047198	*PPARG*
470	epithelial cell migration	36.980392	0.047198	*MMP9*
471	molting cycle process	36.980392	0.047198	*EGFR*
472	hair cycle process	36.980392	0.047198	*EGFR*
473	amyloid precursor protein metabolic process	36.980392	0.047198	*APP*
474	positive regulation of JNK cascade	36.980392	0.047198	*APP*
475	cellular response to acid chemical	36.980392	0.047198	*EGFR*
476	glycolytic process	36.621359	0.047357	*APP*
477	positive regulation of interleukin-6 production	36.621359	0.047357	*APP*
478	cellular response to type II interferon	36.621359	0.047357	*PPARG*
479	chemokine production	36.269231	0.047415	*APP*
480	regulation of chemokine production	36.269231	0.047415	*APP*
481	negative regulation of innate immune response	36.269231	0.047415	*PPARG*
482	positive regulation of lipid localization	36.269231	0.047415	*PPARG*
483	cardiac muscle hypertrophy	35.923810	0.047472	*PPARG*
484	endocrine process	35.923810	0.047472	*PPARG*
485	epithelium migration	35.923810	0.047472	*MMP9*
486	positive regulation of mitotic cell cycle phase transition	35.923810	0.047472	*EGFR*
487	pyridine nucleotide catabolic process	35.584906	0.047625	*APP*
488	ADP catabolic process	35.584906	0.047625	*APP*
489	positive regulation of lipid biosynthetic process	35.584906	0.047625	*PTGS2*
490	negative regulation of cytokine-mediated signaling pathway	34.925926	0.048072	*PPARG*
491	striated muscle hypertrophy	34.925926	0.048072	*PPARG*
492	regulation of neurotransmitter transport	34.925926	0.048072	*APP*
493	purine nucleoside diphosphate catabolic process	34.605505	0.048072	*APP*

	Description	Fold Enrichment	p.adjust	Hub Gene Symbols
494	purine ribonucleoside diphosphate catabolic process	34.605505	0.048072	*APP*
495	defense response to Gram-negative bacterium	34.605505	0.048072	*APP*
496	regulation of binding	34.605505	0.048072	*MMP9*
497	pyridine-containing compound catabolic process	34.605505	0.048072	*APP*
498	regulation of non-canonical NF-kappaB signal transduction	34.605505	0.048072	*APP*
499	long-chain fatty acid metabolic process	34.290909	0.048218	*PTGS2*
500	muscle hypertrophy	34.290909	0.048218	*PPARG*
501	tissue migration	34.290909	0.048218	*MMP9*
502	excitatory postsynaptic potential	33.981982	0.048554	*APP*
503	acute inflammatory response	33.678571	0.048695	*PTGS2*
504	ribonucleoside diphosphate catabolic process	33.678571	0.048695	*APP*
505	ADP metabolic process	33.678571	0.048695	*APP*
506	response to estradiol	33.380531	0.048835	*EGFR*
507	positive regulation of tumor necrosis factor production	33.380531	0.048835	*APP*
508	negative regulation of response to cytokine stimulus	33.380531	0.048835	*PPARG*
509	nucleoside diphosphate catabolic process	33.087719	0.048972	*APP*
510	fibroblast proliferation	33.087719	0.048972	*EGFR*
511	lymphocyte migration	33.087719	0.048972	*APP*
512	macrophage activation	32.800000	0.049053	*APP*
513	negative regulation of monoatomic ion transport	32.800000	0.049053	*MMP9*
514	apoptotic mitochondrial changes	32.517241	0.049053	*MMP9*
515	interleukin-1 beta production	32.517241	0.049053	*APP*
516	regulation of interleukin-1 beta production	32.517241	0.049053	*APP*
517	collagen metabolic process	32.517241	0.049053	*MMP9*
518	regulation of circadian rhythm	32.517241	0.049053	*PPARG*
519	long-term synaptic potentiation	32.517241	0.049053	*APP*
520	positive regulation of canonical Wnt signaling pathway	32.239316	0.049281	*EGFR*
521	positive regulation of tumor necrosis factor superfamily cytokine production	32.239316	0.049281	*APP*

Table S4. Significant enrichment of GO CC terms among the five hub genes associated with TnBP/DBuP-induced trouble sleeping (p < 0.05).

	Description	Fold Enrichment	*p*.adjust	Hub Gene Symbols
1	early endosome membrane	38.170335	0.030698	*APP/EGFR*
2	membrane raft	27.414433	0.030698	*APP/EGFR*
3	membrane microdomain	27.227304	0.030698	*APP/EGFR*
4	endoplasmic reticulum lumen	25.008150	0.030698	*APP/PTGS2*
5	nuclear membrane	24.471166	0.030698	*EGFR/PTGS2*
6	vesicle lumen	23.956757	0.030698	*APP/EGFR*
7	nuclear envelope lumen	362.618182	0.030698	*APP*
8	low-density lipoprotein particle	265.920000	0.033023	*APP*
9	cell leading edge	18.213699	0.033023	*APP/EGFR*
10	early endosome	17.807143	0.033023	*APP/EGFR*
11	very-low-density lipoprotein particle	189.942857	0.033023	*APP*
12	triglyceride-rich plasma lipoprotein particle	189.942857	0.033023	*APP*
13	astrocyte projection	181.309091	0.033023	*APP*
14	high-density lipoprotein particle	147.733333	0.037615	*APP*
15	neuronal dense core vesicle	124.650000	0.038949	*APP*
16	nuclear outer membrane	120.872727	0.038949	*PTGS2*
17	plasma lipoprotein particle	107.805405	0.038949	*APP*
18	lipoprotein particle	107.805405	0.038949	*APP*
19	endosome lumen	104.968421	0.038949	*APP*
20	protein-lipid complex	99.720000	0.038949	*APP*
21	dendritic shaft	94.971429	0.038949	*APP*
22	dense core granule	84.868085	0.040618	*APP*
23	glial cell projection	83.100000	0.040618	*APP*
24	tertiary granule lumen	72.523636	0.043562	*MMP9*
25	nuclear inner membrane	71.228571	0.043562	*PTGS2*
26	platelet alpha granule lumen	59.534328	0.048919	*APP*
27	multivesicular body	58.658824	0.048919	*EGFR*
28	main axon	56.180282	0.049238	*APP*

Table S5. Significant enrichment of GO MF terms among the five hub genes associated with TnBP/DBuP-induced trouble sleeping ($p < 0.05$).

	Description	Fold Enrichment	*p*.adjust	Hub Gene Symbols
1	hormone binding	78.576842	0.024626	*APP/EGFR*
2	carboxylic acid binding	40.133333	0.027907	*APP/PPARG*
3	organic acid binding	39.706383	0.027907	*APP/PPARG*
4	prostaglandin receptor activity	373.240000	0.027907	*PPARG*
5	prostanoid receptor activity	339.309091	0.027907	*PPARG*
6	RAGE receptor binding	311.033333	0.027907	*APP*
7	STAT family protein binding	311.033333	0.027907	*PPARG*
8	icosanoid receptor activity	248.826667	0.027907	*PPARG*
9	acetylcholine receptor binding	248.826667	0.027907	*APP*
10	long-chain fatty acid binding	233.275000	0.027907	*PPARG*
11	acetylcholine receptor regulator activity	219.552941	0.027907	*APP*
12	nuclear retinoid X receptor binding	219.552941	0.027907	*PPARG*
13	apolipoprotein binding	207.355556	0.027907	*APP*
14	neurotransmitter receptor regulator activity	207.355556	0.027907	*APP*
15	heparan sulfate proteoglycan binding	196.442105	0.027907	*APP*
16	insulin receptor binding	162.278261	0.027907	*APP*
17	oxidoreductase activity, acting on single donors with incorporation of molecular oxygen, incorporation of two atoms of oxygen	162.278261	0.027907	*PTGS2*
18	oxidoreductase activity, acting on single donors with incorporation of molecular oxygen	155.516667	0.027907	*PTGS2*
19	WW domain binding	155.516667	0.027907	*PPARG*
20	protein serine/threonine kinase binding	155.516667	0.027907	*APP*
21	nuclear retinoic acid receptor binding	149.296000	0.027907	*PPARG*
22	MAP kinase kinase kinase activity	143.553846	0.027907	*EGFR*
23	low-density lipoprotein particle receptor binding	143.553846	0.027907	*APP*
24	R-SMAD binding	138.237037	0.027907	*PPARG*
25	ephrin receptor binding	133.300000	0.027907	*APP*
26	alpha-actinin binding	133.300000	0.027907	*PPARG*
27	lipoprotein particle receptor binding	120.400000	0.029743	*APP*
28	proteoglycan binding	100.875676	0.032562	*APP*
29	protein tyrosine kinase activator activity	98.221053	0.032562	*EGFR*
30	actinin binding	98.221053	0.032562	*PPARG*
31	frizzled binding	95.702564	0.032562	*APP*
32	chemoattractant activity	86.800000	0.034765	*APP*
33	fatty acid binding	79.412766	0.036832	*PPARG*
34	peroxidase activity	74.648000	0.037335	*PTGS2*
35	oxidoreductase activity, acting on peroxide as acceptor	71.776923	0.037335	*PTGS2*
36	peptide hormone binding	71.776923	0.037335	*APP*
37	nuclear receptor activity	63.261017	0.038418	*PPARG*
38	E-box binding	63.261017	0.038418	*PPARG*
39	ligand-modulated transcription factor activity	62.206667	0.038418	*PPARG*

	Description	Fold Enrichment	*p*.adjust	Hub Gene Symbols
40	transmembrane receptor protein tyrosine kinase activity	61.186885	0.038418	*EGFR*
41	peptidase activator activity	61.186885	0.038418	*APP*
42	collagen binding	51.838889	0.044013	*MMP9*
43	antioxidant activity	48.472727	0.044013	*PTGS2*
44	transmembrane receptor protein kinase activity	47.245570	0.044013	*EGFR*
45	monocarboxylic acid binding	47.245570	0.044013	*PPARG*
46	SMAD binding	46.655000	0.044013	*PPARG*
47	virus receptor activity	46.079012	0.044013	*EGFR*
48	exogenous protein binding	45.517073	0.044013	*EGFR*
49	ATPase binding	41.937079	0.046761	*EGFR*
50	dioxygenase activity	40.133333	0.047865	*PTGS2*

Table S6. Significantly enriched Reactome results of the hub genes ($p < 0.05$).

	Description	Fold Enrichment	p.adjust	Hub Gene Symbols
1	Extra-nuclear estrogen signaling	57.901299	0.034581	*EGFR/MMP9*
2	Signaling by Interleukins	14.138689	0.034581	*APP/PTGS2/MMP9*
3	Interleukin-4 and Interleukin-13 signaling	41.281481	0.034581	*PTGS2/MMP9*
4	G alpha (q) signalling events	20.640741	0.034581	*APP/EGFR*
5	ESR-mediated signaling	20.082883	0.034581	*EGFR/MMP9*
6	PIP3 activates AKT signaling	16.331136	0.034581	*EGFR/PPARG*
7	GRB2 events in EGFR signaling	171.476923	0.034581	*EGFR*
8	Advanced glycosylation endproduct receptor signaling	171.476923	0.034581	*APP*
9	ERBB2 Activates PTK6 Signaling	171.476923	0.034581	*EGFR*
10	SHC1 events in EGFR signaling	159.228571	0.034581	*EGFR*
11	Synthesis of Prostaglandins (PG) and Thromboxanes (TX)	148.613333	0.034581	*PTGS2*
12	Constitutive Signaling by EGFRvIII	148.613333	0.034581	*EGFR*
13	Signaling by EGFRvIII in Cancer	148.613333	0.034581	*EGFR*
14	ERBB2 Regulates Cell Motility	148.613333	0.034581	*EGFR*
15	TFAP2 (AP-2) family regulates transcription of growth factors and their receptors	148.613333	0.034581	*EGFR*
16	Signaling by Nuclear Receptors	14.961074	0.034581	*EGFR/MMP9*
17	Extracellular matrix organization	14.861333	0.034581	*APP/MMP9*
18	GRB2 events in ERBB2 signaling	139.325000	0.034581	*EGFR*
19	PI3K events in ERBB2 signaling	139.325000	0.034581	*EGFR*
20	The NLRP3 inflammasome	139.325000	0.034581	*APP*
21	Signaling by ERBB2 ECD mutants	139.325000	0.034581	*EGFR*
22	Intracellular signaling by second messengers	14.153651	0.034581	*EGFR/PPARG*
23	GAB1 signalosome	131.129412	0.034581	*EGFR*
24	Biosynthesis of DHA-derived SPMs	131.129412	0.034581	*PTGS2*
25	Gastrin-CREB signalling pathway via PKC and MAPK	123.844444	0.034581	*EGFR*
26	Constitutive Signaling by Ligand-Responsive EGFR Cancer Variants	117.326316	0.034581	*EGFR*
27	Nicotinamide salvaging	117.326316	0.034581	*PTGS2*
28	Signaling by Ligand-Responsive EGFR Variants in Cancer	117.326316	0.034581	*EGFR*
29	Biosynthesis of specialized proresolving mediators (SPMs)	117.326316	0.034581	*PTGS2*
30	Signal transduction by L1	106.152381	0.034581	*EGFR*
31	Inflammasomes	106.152381	0.034581	*APP*
32	SHC1 events in ERBB2 signaling	101.327273	0.034581	*EGFR*
33	Deregulated CDK5 triggers multiple neurodegenerative pathways in Alzheimer's disease models	101.327273	0.034581	*APP*
34	Neurodegenerative Diseases	101.327273	0.034581	*APP*
35	Insertion of tail-anchored proteins into the endoplasmic reticulum membrane	101.327273	0.034581	*APP*
36	Signaling by ERBB2 TMD/JMD mutants	101.327273	0.034581	*EGFR*
37	Respiratory syncytial virus (RSV) attachment and entry	96.921739	0.034581	*EGFR*

	Description	Fold Enrichment	p.adjust	Hub Gene Symbols
38	Estrogen-dependent nuclear events downstream of ESR-membrane signaling	92.883333	0.034581	*EGFR*
39	Signaling by EGFR in Cancer	89.168000	0.034581	*EGFR*
40	NOTCH3 Activation and Transmission of Signal to the Nucleus	89.168000	0.034581	*EGFR*
41	TRAF6 mediated NF-kB activation	89.168000	0.034581	*APP*
42	Signaling by ERBB2 KD Mutants	89.168000	0.034581	*EGFR*
43	Defective Intrinsic Pathway for Apoptosis	89.168000	0.034581	*APP*
44	Signaling by ERBB2 in Cancer	85.738462	0.034581	*EGFR*
45	Purinergic signaling in leishmaniasis infection	85.738462	0.034581	*APP*
46	Cell recruitment (pro-inflammatory response)	85.738462	0.034581	*APP*
47	Downregulation of ERBB2 signaling	76.868966	0.036441	*EGFR*
48	SUMOylation of intracellular receptors	74.306667	0.036441	*PPARG*
49	Transcriptional regulation of brown and beige adipocyte differentiation	74.306667	0.036441	*PPARG*
50	Transcriptional regulation of brown and beige adipocyte differentiation by EBF2	74.306667	0.036441	*PPARG*
51	EGFR downregulation	71.909677	0.036441	*EGFR*
52	Nicotinate metabolism	71.909677	0.036441	*PTGS2*
53	Activation of Matrix Metalloproteinases	67.551515	0.038047	*MMP9*
54	Lysosome Vesicle Biogenesis	63.691429	0.039591	*APP*
55	Transcriptional regulation by the AP-2 (TFAP2) family of transcription factors	58.663158	0.04218	*EGFR*
56	Signaling by SCF-KIT	51.841860	0.046836	*MMP9*
57	TAK1-dependent IKK and NF-kappa-B activation	49.537778	0.048137	*APP*
58	Interleukin-10 signaling	47.429787	0.049392	*PTGS2*

Table S7. PDB ID for the proteins associated with hub genes, incorporating the center and docking dimensions. (TnBP and DBuP).

Gene Symbol	PDB ID	Center (x, y, z)	Docking size (x, y, z)
PTGS2	5F19	31.83, 8.25, 59.08	65, 55, 55
PPARG	2Q59	-12.61, 11.644, -17.09	41, 44, 42
APP	8JAL	231.50, 209.99, 223.63	47, 74, 47
EGFR	1M14	22.77, 42.62, 33.29	36, 36, 38
MMP9	1ITV	-40.43, -30.86, -7.26	42, 38, 22

Table S8. Detailed information regarding the AOP network.

	AOP ID	Upstream Event ID	Upstream Event Name	Downstream Event ID	Downstream Event Name
1	Aop: 17	Event: 1538	Decreased protection against oxidative stress	Event: 1392	Oxidative Stress
2	Aop: 17	Event: 1392	Oxidative Stress	Event: 1488	Glutamate dyshomeostasis
3	Aop: 17	Event: 55	Increase, Cell injury/death	Event: 1492	Tissue resident cell activation
4	Aop: 17	Event: 1487	Binding, Thiol/seleno-proteins involved in protection against oxidative stress	Event: 1538	Decreased protection against oxidative stress
5	Aop: 48	Event: 389	Increased, Intracellular Calcium overload	Event: 177	Mitochondrial dysfunction
6	Aop: 3	Event: 887	Inhibition, NADH-ubiquinone oxidoreductase (complex I)	Event: 177	Mitochondrial dysfunction
7	Aop: 12	Event: 55	Increase, Cell injury/death	Event: 188	Neuroinflammation
8	Aop: 3	Event: 890	Degeneration of dopaminergic neurons of the nigrostriatal pathway	Event: 188	Neuroinflammation
9	Aop: 12	Event: 352	N/A, Neurodegeneration	Event: 188	Neuroinflammation
10	Aop: 17	Event: 55	Increase, Cell injury/death	Event: 188	Neuroinflammation
11	Aop: 48	Event: 55	Increase, Cell injury/death	Event: 188	Neuroinflammation
12	Aop: 48	Event: 352	N/A, Neurodegeneration	Event: 188	Neuroinflammation
13	Aop: 13	Event: 201	Binding of antagonist, NMDA receptors	Event: 195	Inhibition, NMDARs
14	Aop: 12	Event: 201	Binding of antagonist, NMDA receptors	Event: 195	Inhibition, NMDARs
15	Aop: 42	Event: 279	Thyroperoxidase, Inhibition	Event: 277	Thyroid hormone synthesis, Decreased
16	Aop: 54	Event: 425	Decrease of Thyroidal iodide	Event: 277	Thyroid hormone synthesis, Decreased
17	Aop: 54	Event: 281	Thyroxine (T4) in serum, Decreased	Event: 280	Thyroxine (T4) in neuronal tissue, Decreased
18	Aop: 42	Event: 281	Thyroxine (T4) in serum, Decreased	Event: 280	Thyroxine (T4) in neuronal tissue, Decreased
19	Aop: 42	Event: 277	Thyroid hormone synthesis, Decreased	Event: 281	Thyroxine (T4) in serum, Decreased
20	Aop: 54	Event: 277	Thyroid hormone synthesis, Decreased	Event: 281	Thyroxine (T4) in serum, Decreased
21	Aop: 12	Event: 352	N/A, Neurodegeneration	Event: 341	Impairment, Learning and memory
22	Aop: 17	Event: 386	Decrease of neuronal network function	Event: 341	Impairment, Learning and memory
23	Aop: 54	Event: 386	Decrease of neuronal network function	Event: 341	Impairment, Learning and memory
24	Aop: 13	Event: 386	Decrease of neuronal network function	Event: 341	Impairment, Learning and memory
25	Aop: 48	Event: 618	Decreased, Neuronal network function in adult brain	Event: 341	Impairment, Learning and memory

	AOP ID	Upstream Event ID	Upstream Event Name	Downstream Event ID	Downstream Event Name
26	Aop: 12	Event: 188	Neuroinflammation	Event: 352	N/A, Neurodegeneration
27	Aop: 48	Event: 55	Increase, Cell injury/death	Event: 352	N/A, Neurodegeneration
28	Aop: 48	Event: 188	Neuroinflammation	Event: 352	N/A, Neurodegeneration
29	Aop: 54	Event: 280	Thyroxine (T4) in neuronal tissue, Decreased	Event: 381	Reduced levels of BDNF
30	Aop: 13	Event: 52	Decreased, Calcium influx	Event: 381	Reduced levels of BDNF
31	Aop: 12	Event: 52	Decreased, Calcium influx	Event: 381	Reduced levels of BDNF
32	Aop: 13	Event: 381	Reduced levels of BDNF	Event: 382	Aberrant, Dendritic morphology
33	Aop: 13	Event: 381	Reduced levels of BDNF	Event: 383	Reduced, Presynaptic release of glutamate
34	Aop: 13	Event: 383	Reduced, Presynaptic release of glutamate	Event: 385	Decrease of synaptogenesis
35	Aop: 13	Event: 382	Aberrant, Dendritic morphology	Event: 385	Decrease of synaptogenesis
36	Aop: 13	Event: 55	Increase, Cell injury/death	Event: 385	Decrease of synaptogenesis
37	Aop: 54	Event: 851	Decrease of GABAergic interneurons	Event: 385	Decrease of synaptogenesis
38	Aop: 13	Event: 385	Decrease of synaptogenesis	Event: 386	Decrease of neuronal network function
39	Aop: 54	Event: 385	Decrease of synaptogenesis	Event: 386	Decrease of neuronal network function
40	Aop: 17	Event: 55	Increase, Cell injury/death	Event: 386	Decrease of neuronal network function
41	Aop: 48	Event: 875	Binding of agonist, Ionotropic glutamate receptors	Event: 388	Overactivation, NMDARs
42	Aop: 48	Event: 388	Overactivation, NMDARs	Event: 389	Increased, Intracellular Calcium overload
43	Aop: 42	Event: 758	Hippocampal Physiology, Altered	Event: 402	Cognitive function, decreased
44	Aop: 54	Event: 424	Inhibition, Na+/I- symporter (NIS)	Event: 425	Decrease of Thyroidal iodide
45	Aop: 13	Event: 195	Inhibition, NMDARs	Event: 52	Decreased, Calcium influx
46	Aop: 12	Event: 195	Inhibition, NMDARs	Event: 52	Decreased, Calcium influx
47	Aop: 17	Event: 1488	Glutamate dyshomeostasis	Event: 55	Increase, Cell injury/death
48	Aop: 17	Event: 1493	Increased Pro-inflammatory mediators	Event: 55	Increase, Cell injury/death
49	Aop: 12	Event: 381	Reduced levels of BDNF	Event: 55	Increase, Cell injury/death
50	Aop: 48	Event: 177	Mitochondrial dysfunction	Event: 55	Increase, Cell injury/death

	AOP ID	Upstream Event ID	Upstream Event Name	Downstream Event ID	Downstream Event Name
51	Aop: 13	Event: 381	Reduced levels of BDNF	Event: 55	Increase, Cell in-jury/death
52	Aop: 17	Event: 188	Neuroinflammation	Event: 55	Increase, Cell in-jury/death
53	Aop: 10	Event: 616	Occurrence, A paroxysmal depolarizing shift	Event: 613	Occurrence, Epileptic seizure
54	Aop: 10	Event: 682	Generation, Amplified excit-atory postsynaptic potential (EPSP)	Event: 616	Occurrence, A paroxys-mal depolarizing shift
55	Aop: 48	Event: 352	N/A, Neurodegeneration	Event: 618	Decreased, Neuronal network function in adult brain
56	Aop: 10	Event: 667	Binding at picrotoxin site, iGABAR chloride channel	Event: 64	Reduction, Ionotropic GABA receptor chlo-ride channel conduct-ance
57	Aop: 10	Event: 64	Reduction, Ionotropic GABA receptor chloride channel conductance	Event: 669	Reduction, Neuronal synaptic inhibition
58	Aop: 10	Event: 669	Reduction, Neuronal synap-tic inhibition	Event: 682	Generation, Amplified excitatory postsynaptic potential (EPSP)
59	Aop: 42	Event: 280	Thyroxine (T4) in neuronal tissue, Decreased	Event: 756	Hippocampal gene ex-pression, Altered
60	Aop: 42	Event: 756	Hippocampal gene expres-sion, Altered	Event: 757	Hippocampal anatomy, Altered
61	Aop: 42	Event: 757	Hippocampal anatomy, Al-tered	Event: 758	Hippocampal Physiol-ogy, Altered
62	Aop: 54	Event: 381	Reduced levels of BDNF	Event: 851	Decrease of GABAergic interneurons
63	Aop: 3	Event: 888	Binding of inhibitor, NADH-ubiquinone oxidore-ductase (complex I)	Event: 887	Inhibition, NADH-ubiquinone oxidore-ductase (complex I)
64	Aop: 3	Event: 177	Mitochondrial dysfunction	Event: 889	Impaired, Proteostasis
65	Aop: 3	Event: 188	Neuroinflammation	Event: 890	Degeneration of dopa-minergic neurons of the nigrostriatal pathway
66	Aop: 3	Event: 889	Impaired, Proteostasis	Event: 890	Degeneration of dopa-minergic neurons of the nigrostriatal pathway
67	Aop: 3	Event: 890	Degeneration of dopaminer-gic neurons of the nigrostri-atal pathway	Event: 896	Parkinsonian motor deficits
